# Deciphering endothelial heterogeneity in health and disease at single-cell resolution: progress and perspectives

**DOI:** 10.1093/cvr/cvac018

**Published:** 2022-02-18

**Authors:** Lisa M Becker, Shiau-Haln Chen, Julie Rodor, Laura P M H de Rooij, Andrew H Baker, Peter Carmeliet

**Affiliations:** Laboratory of Angiogenesis and Vascular Metabolism, Department of Oncology and Leuven Cancer Institute (LKI), KU Leuven, VIB Center for Cancer Biology, VIB, Leuven, 3000, Belgium; The Queens Medical Research Institute, Centre for Cardiovascular Science, University of Edinburgh, Edinburgh EH16 4TJ, UK; The Queens Medical Research Institute, Centre for Cardiovascular Science, University of Edinburgh, Edinburgh EH16 4TJ, UK; Laboratory of Angiogenesis and Vascular Metabolism, Department of Oncology and Leuven Cancer Institute (LKI), KU Leuven, VIB Center for Cancer Biology, VIB, Leuven, 3000, Belgium; The Queens Medical Research Institute, Centre for Cardiovascular Science, University of Edinburgh, Edinburgh EH16 4TJ, UK; CARIM Institute, University of Maastricht, Universiteitssingel 50, 6200 MD Maastricht, The Netherlands; Laboratory of Angiogenesis and Vascular Metabolism, Department of Oncology and Leuven Cancer Institute (LKI), KU Leuven, VIB Center for Cancer Biology, VIB, Leuven, 3000, Belgium; Laboratory of Angiogenesis and Vascular Heterogeneity, Department of Biomedicine, Aarhus University, Aarhus 8000, Denmark; State Key Laboratory of Ophthalmology, Zhongshan Ophthalmic Center, Sun Yat-Sen University, Guangzhou, Guangdong, P.R. China

**Keywords:** Endothelial cell, Heterogeneity, Single-cell OMICs, Vasculature

## Abstract

Endothelial cells (ECs) constitute the inner lining of vascular beds in mammals and are crucial for homeostatic regulation of blood vessel physiology, but also play a key role in pathogenesis of many diseases, thereby representing realistic therapeutic targets. However, it has become evident that ECs are heterogeneous, encompassing several subtypes with distinct functions, which makes EC targeting and modulation in diseases challenging. The rise of the new single-cell era has led to an emergence of studies aimed at interrogating transcriptome diversity along the vascular tree, and has revolutionized our understanding of EC heterogeneity from both a physiological and pathophysiological context. Here, we discuss recent landmark studies aimed at teasing apart the heterogeneous nature of ECs. We cover driving (epi)genetic, transcriptomic, and metabolic forces underlying EC heterogeneity in health and disease, as well as current strategies used to combat disease-enriched EC phenotypes, and propose strategies to transcend largely descriptive heterogeneity towards prioritization and functional validation of therapeutically targetable drivers of EC diversity. Lastly, we provide an overview of the most recent advances and hurdles in single EC OMICs.

## 1. Introduction

Endothelial cells (ECs) line the interior surface of blood and lymph vessels. The endothelium plays a crucial role in maintaining tissue homeostasis in health,^1^ but also contributes to the progression of many diseases.^2^ ECs respond to various physical and chemical stimuli and interact with other cells in the vessel wall, such as smooth muscle cells or pericytes, to regulate vascular tone, blood flow, inflammation, permeability of solutes, and cellular adhesion.^1^ Blood vessel overgrowth promotes diseases like cancer,^3^ while EC dysfunction contributes to vascular complications in diabetes, cardiovascular disease, and ageing-associated pathologies (including neurological diseases with a vascular component, such as Alzheimer’s disease^4^). Hence, understanding the basic function and dysfunction of the endothelium in health and disease has broad reaching implications.

Despite their common characteristics,^5^ ECs are heterogeneous under physiological and disease conditions^6–8^ (see *Box 1* for definition of heterogeneity). Whilst they are present throughout the whole body, ECs are highly specialized to meet the distinct needs of the organs and sites they reside in. Within each organ, this heterogeneity is evident between different vascular beds (arteries, veins, capillaries, and lymphatics), between different segments of the same vessel type, and even between neighbouring ECs.^8^ EC phenotypes in disease are equally diverse, exemplified by their ability to activate or inhibit angiogenesis, metabolic switching or the release of vasodilators, reflecting varying responses to different stimuli and changes in the pathological microenvironment.^3^ While EC heterogeneity was highlighted in other reviews,^6–8^ recent advances in single-cell technologies brought new resolution and new insights into this heterogeneity. Characterizing the different heterogeneity levels and their functional relevance is crucial,^5^ albeit dependent on the technologies used to measure and quantify heterogeneity.^9^

Box 1  What is heterogeneity and how can it be quantified? Heterogeneity is an immanent trait of living systems that is omnipresent across all biological levels. It can manifest in different scales, ranging from different species arising from evolution to genetic differences within a population of seemingly identical cells. Although biological diversity is vital for the survival of organisms in a changing environment, it presents a formidable challenge for biologists to determine which of the observed heterogeneity have a biologically meaningful function. Heterogeneity can be summarized as a statistical characteristic of a cell population. It is most commonly quantified through epigenomic, genomic, transcriptomic, and proteomic studies, though the extent of heterogeneity at one level of regulation is not indicative of the heterogeneity at another level of expression. Conceptually, heterogeneity within a cell population can be probed by first collecting single-cell measurements from the population. Next, patterns of diversity can be identified by distilling distinct cellular behaviours into defined categories. Finally, functional significance of the patterns observed can be tested by measuring whether one subpopulation significantly differs from another or if the heterogeneity is informative as a predictor of responses to certain stimuli. We recommend the following commentaries for further conceptual exploration of heterogeneity in biology and single cell profiling.^5,9^

In this review, after a brief historical perspective on the methods used to study EC heterogeneity, we will focus on novel discoveries regarding EC heterogeneity in health, disease and under therapeutic intervention, made based on single-cell OMICs, and discuss the current challenges and perspectives in the field. Rather than providing an all-encompassing overview, we discuss key principles and examples.

## 2. Historical perspective on methods to unravel EC heterogeneity

Prior to the advent of single-cell technologies, various *in situ* and *in vivo* methods were developed to identify organ- and vessel type-specific endothelial markers whilst circumventing difficulties faced in isolating pure populations of ECs from tissues and the loss of the *in vivo* phenotype of ECs cultured *in vitro* (reviewed in references^10–12^). The concept that ECs from different organs and vascular beds express different molecular markers was fuelled by early evidence of cancer and immune cells, preferentially migrating to specific organs—likewise, peptides with a particular sequence homed to specific vascular beds.^13–16^ For instance, the Stamper-Woodruff assay was designed to study lymphocyte-endothelial binding in lymph nodes,^13^ later modified for use in other tissues,^14,15^ and alongside an emerging monoclonal antibody technology, led to the identification of L-selectin as the receptor responsible for selective homing of lymphocytes to high endothelial venules (HEVs) in lymph nodes.^16^

Phage display peptide libraries were used to unbiasedly screen peptide sequences that home to particular organs^17^ or vascular beds *in vivo*.^18^ These approaches, as well as SAGE analysis^19,20^ and microarrays,^21–23^ contributed to the mapping of endothelial markers across different organs and vascular beds within organs,^24–30^ the development of tissue-targeted pharmacodelivery,^18,31,32^ and to increasing our understanding of baseline EC phenotypes in different organs.^33^ However, these methods suffer from relatively low throughput and parallel processing capabilities, and some of these strategies also require prior knowledge of the cellular states and markers of the subpopulations of interest, limiting their use in identifying novel EC subtypes. In addition, these techniques allow us to study EC heterogeneity only at the bulk, not at the single-cell, level. Hence, the advance in single-cell technologies has had unparalleled influence on the study of ECs.

## 3. Single-cell studies in ECs

**Figure 1:**
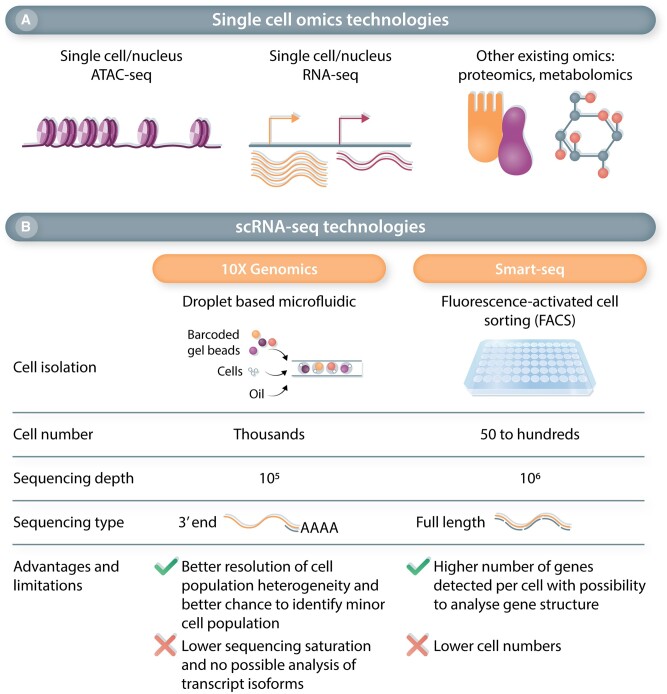
Overview of single-cell omics techonologies and characteristics of the two main scRNA-seq approaches. (*A*) Single-cell OMICs technologies are diverse, profiling different molecules at the single-cell level. scATAc-seq analyses chromatin accessibility while scRNA-seq defines gene expression by measuring RNA steady state level. Other OMICs technologies such as proteomics and metabolomics are less commonly used at the single-cell level. (*B*) Comparison of the two main scRNA-seq technologies in terms of cell isolation, recovered cell number, sequencing depth, and sequencing type. 10× Genomics with its droplet based microfluidics technology allows the sequenting of thousands of cells providing a high resolution of cell populations but without a full coverage of the transcriptome and no information on gene structure. In contrast, SMART-Seq, with its higher sequencing depth and full-length sequencing, provides a better transcriptomics coverage but for a lower number of cells.

Our characterization and understanding of EC heterogeneity have advanced considerably in the past years, due to the development of single-cell OMICs approaches (*Figure 1A*). These techniques offer simultaneous analysis of hundreds to thousands of cells from complex samples, such as tissues and heterogeneous cell populations, often without any prior knowledge of cell markers.^34^ In particular, single-cell transcriptomics have been used to identify and study EC populations in health and disease, across virtually all stages of life (e.g. development, adulthood, ageing), as detailed hereafter. While initial studies often described a single organ in healthy condition,^35–40^ recent studies now provide multi-organ analysis^41–44^ or focus on a specific disease, allowing to study ECs in physiological and pathological conditions. Most single-cell RNA-sequencing (scRNA-seq) experiments relied on a droplet-based approach, with the majority using the 3′ end sequencing Chromium 10× technology. The main characteristics and advantages of the two major scRNA-seq platforms (10× Genomics and Smart-Seq) are described in *Figure 1B*, and more details on these technologies and applications can be found in several reviews.^45,46^ Some scRNA-seq studies were also accompanied by single-cell ATAC-seq (Assay for Transposase-Accessible Chromatin sequencing), revealing the (epigenetic) chromatin accessibility landscape in ECs.^47–50^ scRNA-seq studies of ECs using mouse tissues/models took advantage of tissue availability, allowing a more in-depth study of development and/or early disease stages, for which only late-stage human disease samples are available, such as pulmonary arterial hypertension (PAH).^51^ scRNA-seq was also performed in healthy and diseased human tissues, such as types of lung,^52–58^ liver,^59^ heart,^60–63^ or brain^64^ diseases. Only a few studies combined both human and mouse analysis,^53,65,66^ allowing a cross-species comparison. Of note, the interpretation and findings of the EC scRNA-seq studies described below are also limited by the study design and chosen data analysis pipelines. Some of these limitations, as well as the general caveats of scRNA-seq analyses, have been highlighted in *Box 2*.

Box 2  Study design and bioinformatics consideration for scRNA-seq studies to identify and characterize EC populations on the transcriptome level *Whole tissues vs EC enrichment vs EC isolated from reporter mice*: Whole tissue/organ analysis potentially lowers the power and resolution of EC analysis, yet allows their analysis amidst other cell types and querying of cell-cell interactions. To obtain a better resolution of the EC transcriptomic landscape, enrichment strategies based on CD31 expression can be performed prior to sequencing.^41,65,97,102^ scRNA-seq of ECs isolated from reporter mice have also been implemented in liver cirrhosis,^100^ after myocardial infarction^89,103^ and in atherosclerosis.^197^ Such designs allow the tracking of changes that ECs undergo in diseases and reveal the presence and/or absence of cell transitions, such as endothelial-to-mesenchymal transition (EndMT).^103^

*Cell number*: A low number of sequenced cells could limit the identification of minor EC populations.

*Inclusion of technical and biological replicates*: As expected for novel technologies and in part due to their costs, study designs vary considerably in terms of biological/technical replicates. In some studies, lack of or a low number of replicates prevent an analysis of variability and reproducibility and will require further studies and additional validations.

*Depth of EC downstream analysis*: The depth of downstream EC analyses also varies across different studies, sometimes due to the study design (e.g. limited number of isolated ECs), or to incomplete characterization of EC clusters. Especially in cases of whole tissue scRNA-seq, EC analysis has often been performed alongside the analysis of other more abundant cell types, and lacks in-depth investigation and/or detailed subclustering of ECs. For instance, in studies of abdominal aortic aneurysm,^198^ Alzheimer disease,^199^ cancer,^56^ cirrhosis/fibrosis,^52,59^ and atherosclerosis,^200^ ECs were present but their EC subsets were not studied.

*scRNA-seq analysis- general caveats*: Besides EC-specific considerations in terms of study design and analysis, hurdles in quality control (QC) of the data remain an ongoing challenge in the field of single-cell OMICs. For instance, during library preparation using droplet-based methods, multiple cells may have been captured together (doublets), non-viable cells may have been captured, or, droplets may have been sequenced that harboured no cells (empty droplets). Differences in library preparation might also stem from variability in cell recovery and quality, which results from different isolation protocols. After sequencing, it is thus imperative to implement a series of QC steps to ensure the analysis will be performed on high-quality cells only. Generally, QC of scRNA-seq data is based on three variables: (i) the number of counts per cell, (ii) the number of genes per cell, and (iii) the fraction of counts from mitochondrial genes per cell. Filtering of outliers, based in examination of the distributions of these QC variables, can be applied to eliminate unwanted cells. For example, low-quality cells can be identified by a low number of detected genes, non-viable cells are characterized by a high fraction of mitochondrial counts, and cells with an unexpectedly large number of detected genes may represent doublets. Specifically for doublet removal, several computational tools can additionally be used to further optimize their detection beyond manual inspection of gene counts (DoubletDecon,^201^ Solo,^202^ scds,^203^ Scrublet,^204^ and Doublet Finder^205^). Additionally, cell hashing strategies can be implemented to enhance the detection of doublets.^206^

scRNA-seq results typically also suffer from sparsity, as the data often only captures a small fraction of the transcriptome, and genes can be detected at a low or moderate expression level in one cell, yet go undetected in another cell of the same cell type (zeros). Several computational approaches can be implemented to tackle this problem. Selecting only the most highly variable genes in the data, and applying several dimensionality reduction strategies represent common methods of handling data sparsity.^207^ Moreover, various methods have been developed to ‘impute’ values for observed zeros, including SAVER^208^ and MAGIC.^209^

Furthermore, to accurately decipher findings from scRNA-seq data, normalization is an essential step to adjust for unwanted biases resulting from sequencing depth, sparsity, and other potential technical artefacts. Numerous normalization methods have been developed specifically for scRNA-seq data. One of the most general methods of normalization is the NormalizeData function, implemented within the Seurat R package. With this method, gene counts for each cell are normalized by the total expression, before multiplying by the scale factor (10 000 by default) and natural log transforming the result. Various alternative normalization methods have been described and tested, but these appear highly comparable to the method built in to Seurat.^210^

## 4. Endothelial heterogeneity in health

In the healthy adult, phenotypic and structural diversity of ECs are a reflection of the breadth of functions they perform to maintain tissue homeostasis and are highly dependent on the organs and microenvironment in which they reside. Given that different organs have different needs,^8^ dissecting the functional specialization of ECs in healthy organs is key to understanding EC health and behaviour, and essential for identifying how and why they become dysfunctional in disease.

### 4.1 Organotypic heterogeneity

**Figure 2:**
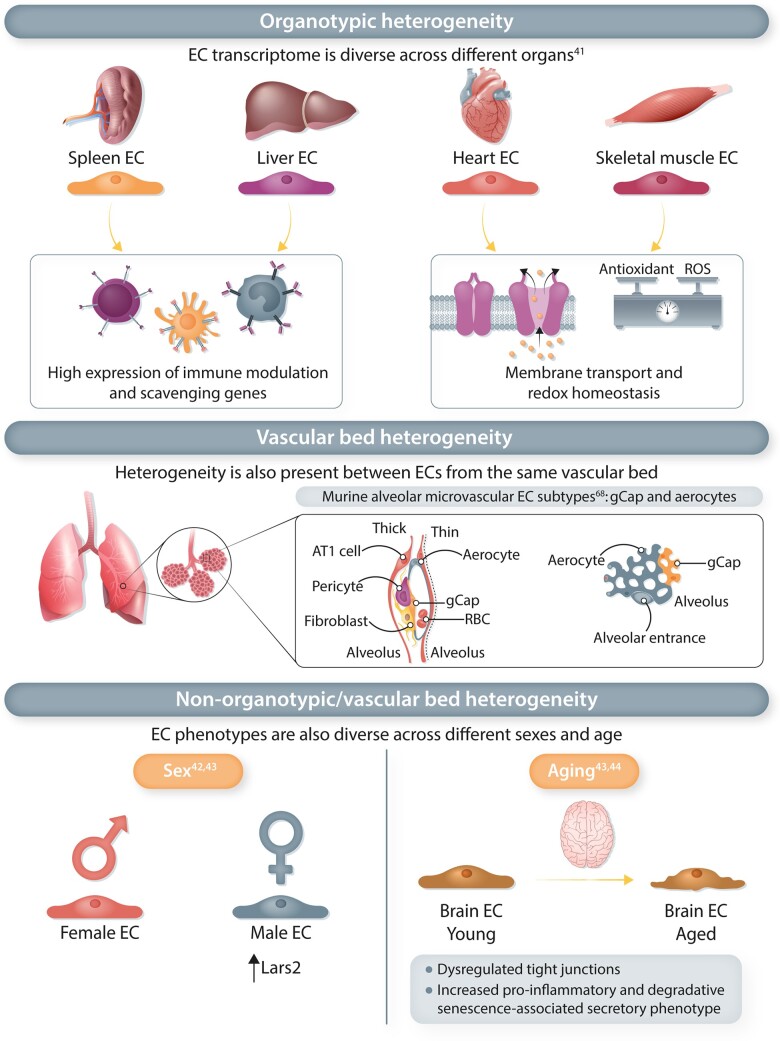
Endothelial heterogeneity in health. EC phenotypes in health differ across organs, vascular beds and non-organotypic/vascular bed factors including sex and ageing. *Organotypic heterogeneity*: ECs from different organs highly express genes involved in different biological processes. Liver and spleen ECs have a shared high expression of gene sets involved in immunoregulation, whilst heart and skeletal muscle ECs have up-regulated expression of genes associated with membrane transporter and redox homeostasis. *Vascular bed heterogeneity*: Within each vascular bed, ECs from different segments of the same vessel type are diverse with several different EC subtypes. Two different subtypes of murine lung capillary ECs have been identified, aerocytes and general capillary ECs (gCap). Modified illustration from Gillich *et al*.^68^*Non-organotypic/vascular bed heterogeneity*: EC phenotypes also vary across sex and age. Male ECs have enriched *Lars2* expression, compared to female ECs. Aged ECs are phenotypically different from younger ECs, such as brain capillary ECs expressing more pro-inflammatory and senescence-associated genes, resulting in dysregulated tight junctions in the blood–brain barrier.

Initial single-cell transcriptomic studies characterizing EC heterogeneity focused largely on single tissues.^35,36,39,67^ Although these studies highlighted organotypic diversity in EC populations, and increased our understanding of the EC subtypes in individual organs, a robust comparison across different tissues requires multi-organ studies. A recent study performed scRNA-seq on 32 567 ECs from 11 different murine organs to create a comprehensive single-EC transcriptome atlas (EC atlas).^41^ Across different organs, inter-tissue heterogeneity in EC transcriptional states was detected. ECs from different organs expressed distinct transcriptional signatures, although ECs from certain organs had overlapping gene signatures, suggesting shared biological processes.^41^ Higher expression of gene sets involved in immune modulation and scavenging were for example shared by liver and spleen ECs, while an enrichment of genes involved in membrane transport was detected in heart and skeletal muscle ECs^41^ (*Figure 2*). Different transcription factor (TF) networks were moreover up-regulated in ECs from different tissues, which may drive organotypic diversity of ECs. Regulons of the *Gata* family were for example enriched in liver and spleen ECs, while spleen ECs additionally showed up-regulation of *Nr5a1* (*Figure 2*). Skeletal muscle ECs displayed enriched expression of the *Pparg* network, while pulmonary ECs showed higher expression of the *Foxf1* network. Another multi-organ study extracted mouse EC transcriptomes across 12 different organs from the single-cell dataset generated by the Tabula Muris consortium.^42^ Largely similar overall findings were reported in both studies regarding unique EC molecular profiles across different organs and overlapping gene expression between certain organs, as well as enrichment of similar gene sets in the same organs (e.g. up-regulation of transporter-related genes in brain ECs).

### 4.2 Vascular bed heterogeneity

Apart from organotypic heterogeneity of ECs, endothelial diversity also exists within the vascular bed (artery, vein, capillary, and lymphatic). In the aforementioned multi-organ study,^41^ conservation of EC vascular diversity across different organs was reported, as arterial, venous, capillary and lymphatic ECs clustered together, regardless of the organ they originated from. The vasculature in all organs displayed an arteriovenous hierarchy and the topography of various endothelial subclusters along the vascular tree paralleled differences in blood flow, pressure, and chemical composition in the circulation.^41^

scRNA-seq allowed finetuning of the traditional blood vascular EC classification (artery, capillary, and vein). For instance, 24 renal endothelial populations were identified across the glomerular, cortical, and medullary compartments,^37^ whilst studies on pulmonary ECs highlighted extensive heterogeneity within the capillary endothelium.^68,69^ The murine alveolar microvasculature was reported to consist of two cellular subtypes, aerocytes and general capillary ECs (gCap), both of which were morphologically distinct from other capillary cells in the bronchial circulation and other organs.^68^ Aerocytes with large, thin, and expansive morphology, are anatomically localized with alveolar type I (AT1) cells and enriched with adhesion and leucocyte-sequestration genes, suggesting that these capillary ECs are unique to the lungs and are specialized for optimal gas exchange and leukocyte trafficking (*Figure 2*). In contrast, gCap cells are positioned in thick regions of the pulmonary stroma, regulate vasomotor tone, and function as specialized stem/progenitor cells in alveolar capillary homeostasis and repair. Both these alveolar capillary EC subtypes and their subtype-specific functions are conserved in humans, although human aerocytes express major histocompatibility complex (MHC) class II genes whilst in mice, these genes are preferentially expressed by gCap cells.^68^ scRNA-seq of human pulmonary cells also identified nine subpopulations of ECs, including two bronchial endothelial groups that were distinctly enriched in matrix, fenestration and cell cycle-related genes, compared to ECs that make up the pulmonary circulation^69^ (*Figure 2*). In addition, there were also two rare capillary subpopulations with features of both aerocytes and gCap cells.

One of the first studies to use scRNA-seq to systematically investigate the molecular profiles of vascular cells in the adult mouse brain identified gradual changes in endothelial transcriptional profiles along the arteriovenous axis, known as zonation.^70^ While clusters of cells corresponding to arterial, microvascular and vein ECs could be identified, these cells could be ordered into a single one-dimensional range with markers of the different clusters displaying a gradual change across this axis. Arterial ECs were enriched in TFs, whilst transporter transcripts were dominantly expressed in capillary and vein ECs, suggesting that trans-endothelial transport of molecules across the blood–brain barrier (BBB) are concentrated in the latter regions. Similar zonation was observed in liver sinusoids, with 67% of liver sinusoidal ECs asymmetrically distributed along the portal vein-central vein axis, though there was limited conservation of zonation profiles between human and mouse scRNA-seq data.^38–40^ These studies provided insights into how zonation influences endothelial function and have implications for improving central nervous system drug delivery in treating brain diseases, as the BBB remains a significant physiological hurdle for drug design and development,^71^ and for understanding the relevance of EC zonation in disease pathogenesis.

### 4.3 Non-organotypic/vascular bed heterogeneity

Apart from organotypic and vascular heterogeneity, endothelial phenotypes were also found to differ between gender and ages in normal health.

#### 4.3.1 Gender

Since the Tabula Muris consortium used both male and female mice, their single-cell studies allow for the assessment of gender as a potential factor contributing to transcriptome diversity among ECs from the same organ. Indeed, adult male and female mice showcase different endothelial gene expression signatures and subpopulations in the brain, heart, and lung.^42^ For instance, the gene encoding mitochondrial leucyl-tRNA synthetase (*Lars2*) is enriched in male vs. female ECs (*Figure 2*). Another study using the endothelial compartment from the same Tabula Muris dataset did not find any differences in EC subtype abundance between male and female mice, though this particular study had used another EC annotation method (scmap and top 10 marker genes of each EC phenotype) to map the Tabula Muris-derived ECs onto the subpopulations identified in their EC Atlas.^41^ However, this study did not further examine EC sex differences beyond the comparison of subtype proportion. A third study, using an independent Tabula Muris dataset generated from young and aged mice (3 months and 18 months),^43,44^ found similar up-regulation of *Lars2* in the young male mice in addition to the up-regulation of S100a8 and S100a9 in the older male mice, when compared with the female. However, they concluded that EC gene expression was largely similar between the sexes when taking age into consideration. Further investigation is warranted to reveal how gender influences EC heterogeneity, and may explain gender differences in cardiovascular risks.

#### 4.3.2 Ageing

Natural ageing influences changes in endothelial phenotypes and may explain age-related susceptibility to diseases.^72^ In an attempt to uncover the impact of ageing on the mammalian heart, one study compared the single-cell transcriptomes of cardiac cells from 12-week-old and 18-month-old mice.^73^ Findings from this study suggest that the paracrine crosstalk between cardiac fibroblasts and cardiac ECs is impaired during ageing. Blunted angiogenesis and autophagy, as well as proinflammatory activation in aged cardiac ECs were attributed to aged fibroblasts, which had the most significant differential gene expression. Increased expression of serpins in aged fibroblasts was found to mediate the anti-angiogenic effects on cardiac ECs. A separate study investigating how ageing affects neurovascular dysfunction compared single endothelial transcriptomes from young (2–3 months old) and aged (18–20 months old) mouse brains.^74^ The age-associated transcriptional changes were involved in immune/cytokine signalling (*Arhgap5, Pak2, Rdx, Gng5, Cdkn1a, Hnrnpk*), BBB integrity (*Afdn, Ctnna1, Iqgap1, Cgnl1, Nedd4, Ocln*), and energy metabolism (*Cox6c, Cox7b, Ucp2, Hmgcs2, Pea15a*), most prominently in capillary ECs. Another study observed up-regulation of von Willebrand factor, a marker of endothelial dysfunction, in gCap cells but not aerocytes, in the lungs of aged mice.^68^ ECs across five different organs in aged mice (18 months) have higher expression of immune and inflammation-related genes, compared to their younger counterparts.^43^ Taken together, these findings suggest heterogeneous regulation of the different EC populations during ageing that may contribute to the development of chronic diseases such as atherosclerosis, hypertension and Alzheimer’s disease (*Figure 2*).

### 4.4 Endothelial heterogeneity in development

EC functional heterogeneity during development is evident in the developing heart,^75^ where the endocardium, a specialized endothelium lining the inner heart walls, acts not only as a physical barrier protecting the cardiac tissue from the chamber circulation but also as an essential source of different cardiac cell types.^76^ Heart valve formation begins with the development of endocardial cushions at the atrioventricular canal and outflow tract, and at E8.5 to 9.0, a subset of these cushion endocardial cells undergoes endothelial-to-mesenchymal transition (EndMT) to give rise to the precursor cells that will eventually go on to form the mature heart valves.^77^ Previously, it was unknown if this endocardial subset was predetermined to undergo EndMT or if the surrounding myocardium and haemodynamic circulation push this subset towards such a fate, since the trabeculae endocardium does not undergo EndMT. Endocardial heterogeneity was confirmed by a recent scRNA-seq study, which sequenced 36 000 cardiac cells from three distinct developmental stages at E7.75 when cardiac progenitor cells begin to differentiate, during heart tube formation at E8.25 and at E9.25 when the heart tube loops.^75^ This study identified three endocardial subpopulations: haematoendothelial progenitors, ECs and endocardial cells initiating EndMT. However, this study did not further examine these subpopulations beyond their identification and assignment in the single-cell dataset. As such, important questions remain about the origin(s) of endocardial subpopulations and the wider endothelial heterogeneity in vascular development: (i) Are all ECs different from the initial point of their formation; and (ii) If not, when do they start becoming different and what drives this differentiation during development?

Since a functioning circulatory system is vital for embryonic growth, formation of the vascular network precedes the formation of all other organ systems. ECs originate *de novo* by vasculogenesis from mesodermal precursors in at least three sites: the yolk sac, allantois, and embryo proper. Primitive ECs at this stage are highly plastic and were presumed to be non-specialized as they undergo rapid expansion and coalesce to form the primary vascular plexus, before acquiring arterial, venous and lymphatic identities. A scRNA-seq of whole mouse embryos at E8.25 reported that subsets of these primitive ECs show unique identities that could be demarcated by their maturity and anatomical origins.^78^ Allantoic ECs express distinct transcriptional signatures, characterized by *Tbx4, Hoxa10*, and *Hoxa11* expression, while non-allantoic ECs could be subdivided by their maturity based on their expression levels of *Etv2, Cdh5*, and *Pecam1*. These findings, alongside scRNA-seq profiling of early Xenopus embryos,^79^ suggest that EC diversity begins much earlier in development than previously thought. It remains to be seen if and how this early diversification of EC identity influences their heterogeneous function and phenotypes later in life, and in the pathophysiology of diseases.

As the vascular plexus continues to remodel into distinct vasculatures, developing ECs continue to differentiate into the different vessel types and subsequently specialize to meet the needs of their resident organs during organ vascularization. Bipotentiality has been reported in pulmonary plexus cells, as they give rise to both subsets of alveolar capillary ECs (aerocytes and gCap cells) during development.^68^ Aerocyte development has also been reported to depend on AT1-derived Vascular Endothelial Growth Factor A (VEGF-A), as this population of ECs is specifically and completely lost in AT1-specific *Vegfa* mutant lungs.^80^ These findings again suggest early specification of EC phenotype during development that continues to persist in the adult.

Lineage-tracing and time-lapse imaging studies provided evidence that a subset of primitive ECs, termed hemogenic ECs, give rise to haematopoietic stem and progenitor cells (HSPCs) and intra-aortic haematopoietic clusters in the later (definitive) wave of haematopoiesis.^81,82^ It is less well-defined if hemogenic ECs are responsible for the primitive wave, where blood cell production occurs in blood islands in the yolk sac, prior to initial vascular formation. This is largely due to the overlap in their cell surface marker expression with haematopoietic cells, though previous studies have shown that the primitive wave can arise from cells expressing endothelial markers *Tie2, VE-cadherin*, and *Pecam1.*^83^

A pseudotemporal dataset of the developing mouse embryo was generated through scRNA-seq from nine sequential timepoints, E6.0 to E8.5.^50^ This study identified two discrete subsets of hemogenic ECs, expressing both endothelial and haematopoietic markers. One of the subpopulations showed a more mature EC phenotype, with a high expression of classical markers of mature ECs such as *Cdh5* and *Pecam1*. By incorporating temporal information of each individual cell, this group was identified as the hemogenic ECs involved in the definitive wave, suggesting that EC maturity is essential to give rise to HSPCs. In addition, they also observed that these second wave ECs were transcriptionally heterogeneous, and through clustering analysis, this heterogeneity was associated with their anatomical origins. This study also reported TAL1 as a transcriptional regulator of the two haematopoietic waves, and documented that *Tal1*^*−/−*^ ECs deviate into an aberrant mesodermal phenotype. An additional study using ATAC-seq on single nuclei from 10 mouse embryos at E8.25 identified EC-specific regions of open chromatin.^84^ Integrative analysis with TAL1 ChIP-seq data from past studies and validation in transgenic mouse assays revealed that TAL1 binds to both known (*Fli* −15 kb and *Erg* +86 kb) and novel (*Flt1* + 67 kb and *Malm3* + 360 kb) endothelial enhancers. Altogether, important transcriptomic and epigenetic mechanisms direct ECs towards a hemogenic fate during development.

## 5. Endothelial heterogeneity in disease

### 5.1 EC population shifts in disease

**Figure 3:**
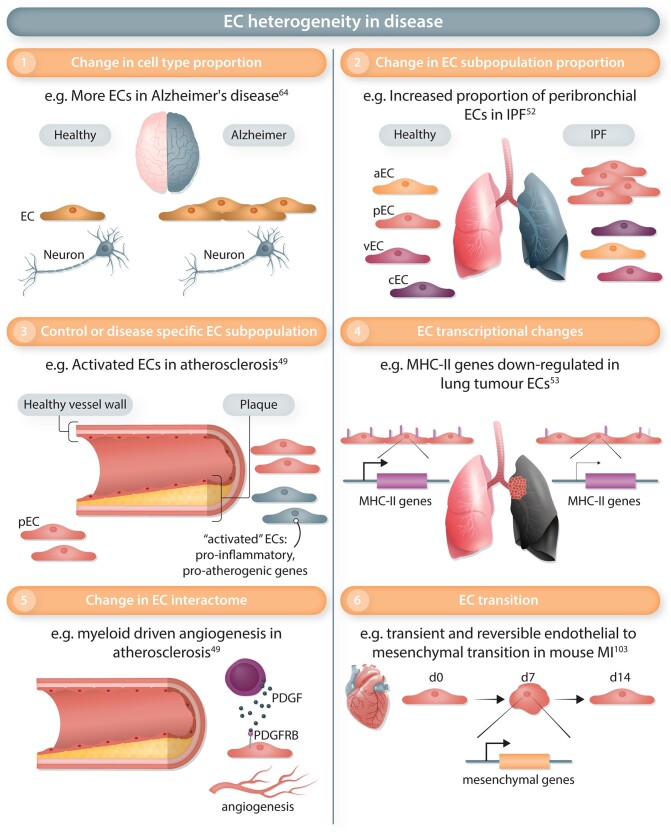
Endothelial heterogeneity in disease. ECs in a pathological context can differ from those in healthy organs on several levels. Each level of heterogeneity highlighted in this figure has been illustrated by a representative example. (1) The relative proportion of ECs (out of all cell types) can change in disease, for example, with more ECs observed in brain tissues from Alzheimer's disease patients compared to other cell types. (2) Disease can trigger a change in the relative proportion of EC subtypes such as an increased abundance of peribronchial ECs (pEC) but not arterial, vein, and capillary ECs (aEC, vEC, and cEC) in idiopathic pulmonary fibrosis (IPF). (3) Specific EC subpopulations can be specifically observed in control or disease conditions. For instance, ‘activated’ ECs expressing pro-inflammatory and pro-atherogenic genes were observed in human atherosclerotic plaques. (4) Disease-mediated transcriptional changes constitute an additional level of heterogeneity. Genes involved in the major histocompatibility complex of class II (MHC-II) are down-regulated in lung tumour ECs. (5) In diseases, ECs can change their interactions with neighbouring cell types. In atherosclerotic plaques, an increased interaction was observed between myeloid cells and ECs mediated by Platelet Derived Growth Factor (PDGF)/Platelet Derived Growth Factor Receptor β (PDGFRB) and leading to angiogenesis. (6) ECs can transition to another cell type by losing their EC markers and gain other cell type identity markers. In mouse, the transient activation of mesenchymal genes has been observed 7 days after myocardial infarction (MI).

Dimensionality reduction and clustering analysis allowed the comparison of EC populations in disease samples. First, a change of the relative proportion of ECs compared to other cell types has been noted in some diseases (*Figure 3*), with for example fewer ECs detected in metastasis compared to primary tumours,^85^ while more ECs have been observed in Alzheimer’s disease vs. control samples.^64^

Within the EC population, a change in the proportion of EC subtypes corresponds to a second level of heterogeneity observed in disease (*Figure 3*). Expansion of one of the three EC subtypes, probably corresponding to post-capillary venular cells, was observed in human skin samples from patients with atopic dermatitis or psoriasis.^86^ In idiopathic pulmonary fibrosis (IPF), the peribronchial EC population was increased compared to control or obstructive pulmonary disease conditions and associated to areas of bronchiolization and fibrosis, showing the distinct response of this population between two diseases.^52^ In mouse lungs exposed to hyperoxic conditions, an increase of the aerocytes/*Car4^+^* ECs population was observed.^87^

An increase in EC proliferation was previously associated with several diseases^88^ and scRNA-seq showed evidence of such an increase after myocardial infarction (MI)^89^ or H1N1 influenza lung injury^90^ in mice. In the lungs, most vessel-type ECs contribute to the proliferating response,^90^ while, in the MI study, the use of a Platelet Derived Growth Factor Subunit B (PDGFB)-driven multispectral (Confetti reporter) EC tracing mouse model confirmed that proliferating ECs originated from resident cells via clonal expansion.^89^ This Confetti reporter mouse line system was previously used to show EC clonal expansion after ischaemia-induced neovascularization, and clonally expanded ECs selected by laser capture microscopy were analysed by bulk transcriptomics without single-cell resolution.^91^ scRNA-seq was also used to study EC populations contributing to liver^92^ and aorta^93^ regeneration after injury in mice. In liver injury, a tissue-resident *Cd157^+^* population contributes to the regeneration of large vessels expressing only EC-specific genes.^92^ In the aorta, regeneration originates from local adjacent ECs; both bulk and scRNA-seq studies revealed transcriptomic changes, including an increase of the progenitor marker *Ly6a*/*Sca1* and the transcription factor (TF) *Aft3*.^93^

Disease can lead to a third level of heterogeneity in the endothelium, with the presence of EC subpopulations being almost exclusively restricted to control or disease conditions (*Figure 3*). After MI in mice, several clusters were predominantly composed of cells from disease samples and were characterized by a higher expression of the plasmalemma vesicle-associated protein gene *Plvap*,^89^ shown to regulate EC proliferation *in vitro*^89^ and previously involved in EC permeability and angiogenesis.^94^ In human liver cirrhosis, two disease-specific EC populations restricted to the fibrotic niche were identified and annotated as scar-associated ECs, in which marker gene analysis revealed the expression of pre-fibrotic and immune response genes.^59^ Furthermore, pro-inflammatory and pro-atherogenic genes characterized EC clusters from the mouse aorta exposed to disturbed flow.^48^ Similar pathways seem to be identified in ECs from human atherosclerotic plaques, in which atherosclerosis-specific EC populations were described as activated ECs.^49^ The term ‘activated ECs’ was also used to describe EC populations identified in prostate cancer, which express cancer-associated fibroblast markers and extracellular matrix (ECM) genes but show a down-regulation of genes related to immunoregulatory pathways.^95^

Tip ECs are critical for vessel sprouting, by leading the sprout at the forefront.^96^ In both human and mouse lung tumours, tip EC populations have been detected in scRNA-seq studies, in agreement with the role of angiogenesis in tumour growth and proliferation.^53,65^ Proliferating cells were detected, at substantial rates in mouse tumours, but at negligible rates in human (lung) tumours.^53^ Tip cells were also found in scRNA-seq studies of mouse choroidal neovascularization.^65^ Common/congruent tip cell markers, conserved across species (mouse/human), diseases and tissues (cancer/choroidal neovascularization), and experimental conditions (freshly isolated/cultured) were identified, allowing a better understanding of angiogenesis across disease conditions.^53,65^ Congruent tip cell markers included genes previously detected in tip cells, such as *APLN*, but also novel tip cell TFs *TCF4, SOX4*, and *SMAD1*, and novel genes relevant to the migratory tip EC phenotype.^53^ Silencing of two novel markers, LXN (*Latexin*) and FSCN1 (*Fascin*), in human umbilical vein ECs furthermore affected tip cell competitivity in a mosaic spheroid assay, confirming the tip cell role of these markers.^53^ In addition to tip cells, another population of so-called ‘breach’ cells has recently been identified in murine lung tumours by scRNA-seq. Based on their transcriptional profile breach cells are hypothesized to assist tip cells to lead the vessel sprout.^53^ In addition, transitioning populations and pseudotime trajectories leading to these tip cells were characterized, revealing a change in the expression of genes related to metabolic pathways.^65^ Such metabolic changes in ECs, key to angiogenesis, were previously reported in scRNA-seq of all cells from lung cancer.^55^ Moreover, in mouse cerebral cavernous malformations, based on a *Pcd10* deletion model, ECs with tip cell traits have been reported^97^ but further characterization is required to confirm if they indeed represent genuine tip cells.

EndMT occurs in many cardiovascular diseases,^98^ yet with some controversies due to the lack of standard in diagnosing the transition, and difficulties comparing different time points and/or models.^99^ Using scRNA-seq of EC reporter mice, no evidence of EndMT was found in liver cirrhosis.^100^ In contrast, EndMT was reported in human calcific aortic valve disease,^101^ in human atherosclerosis^49^ and in mouse atherosclerosis induced by disturbed flow^48^ or the high-cholesterol high-fat diet in Apoe^−/*−*^ mice.^102^ However, these scRNA-seq studies reporting EndMT did not use an EC tracing system, not allowing the full confirmation of the transition, and relied essentially on trajectory analysis. Additional analysis, such as RNA velocity might help to define the directionality of the observed trajectories and the cell population origins. Recently, activation of ECM genes was observed 7 days after MI in the mouse, and confirmed in scRNA-seq analysis of an EC lineage tracing model.^103^ This study, based on a time course experiment, showed that EndMT is transient and reversible in MI,^103^ in contrast to the sustained EndMT observed in atherosclerosis and likely due to the chronic nature of the stimuli.^48,49,102^ The potential transient nature of EndMT might explain why EndMT was not detected in another MI mouse study^89^ and highlights the need to study different stages of disease development in association with a better EndMT diagnosis.^98^

### 5.2 Transcriptomics changes leading to EC heterogeneity

In addition to a change of the population landscape, scRNA-seq also revealed EC global and subtype-specific transcriptomics changes in disease, highlighting a heterogeneity of phenotypes (*Figure 3*).

Changes in genes related to inflammation have been observed in ECs in several contexts. In the adult mouse, peripheral lymph nodes, antigenic stimulation by oxazolone led to an up-regulation of inflammatory genes such as *Sele* and *Cxcl9* in HEVs.^104^ In mouse hyperoxic lungs, genes known to be regulated by inflammation (*Ctgf, Fxyd5*) were up-regulated in the aerocyte EC populations.^87^ In Alzheimer’s diseases, up-regulation of genes from the MHC class I were observed in ECs,^64^ while the expression of the MHC class II genes, part of the capillary gene signature, are up-regulated in PAH^105^ and down-regulated in ECs from murine and human lung tumours.^53^ Changes in inflammation-related genes were also reported in atherosclerotic Apoe^*−*/*−*^ mice,^102^ and a recent study of the mouse aorta during disturbed flow suggested a potential transition of ECs towards an immune-like phenotype as an additional type of EC reprogramming.^48^ All these studies confirm that the endothelium is a target of the inflammatory process, but likely also acts as an immuno-regulator, in part by working as semi-professional antigen-presenting cells. Indeed, the term ‘immunomodulatory ECs’ (IMECs) was recently coined to describe the immunoregulatory EC phenotype.^106^

Vessel growth dysregulation contributes to the pathogenesis of many diseases such as cancer and PAH. In addition to the identification of angiogenic tip cells, angiogenesis pathway regulation has also been documented in several studies. Indeed, down-regulation of genes relevant to capillarization were observed in ECs in human systemic sclerosis,^107^ while anti-angiogenic genes were up-regulated in ECs from hyperoxic lungs.^87^ In contrast, pro-angiogenic/capillarization genes were activated in ECs in Alzheimer’s disease^64^ and cirrhotic mouse liver^100^ and in one capillary EC subtype in PAH.^105^ Interestingly, in cirrhotic liver, the activation was zonation-dependent and restricted to a specific region of the liver sinusoidal ECs.^100^ As most changes of angiogenesis pathway did not seem to be associated with the detection of a tip cell population, these regulations might not be linked to sprouting angiogenesis (SA) but might possibly reflect other vessel formation modes such as splitting angiogenesis, not characterized so far by any standard marker expression, or EC migration. Further studies are needed to understand the contribution of these different processes to vessel growth or regression.

Several studies reported the up-regulation of ECM genes in ECs in disease conditions, probably reflecting structural EC changes. In prostate cancer, activated ECs were characterized by an up-regulation of ECM genes,^95^ while the transient mesenchymal gene activation in MI also included ECM gene changes. In addition, ECM gene up-regulation was observed in liver cirrhosis,^100^ lung cancer^53^ and in systemic sclerosis.^107^ Additional transcriptome regulations in ECs have also been described. Down-regulation of several members of the Notch signalling pathway occurs in ECs in pulmonary fibrosis.^57^ In atopic dermatitis and psoriasis, ECs activate foetal genes,^86^ while in oxygen-induced retinopathy, the peak of neovascularization was associated with expression of senescence genes.^108^ Further investigation is required to define the functional effect of these changes and their relevance across diseases.

To understand the regulation leading to these transcriptomics changes, some scRNA-seq studies were performed together with single-cell ATAC-seq, confirming chromatin accessibility changes in correlation with the transcriptomics changes and reporting disease-induced peaks such as in mouse MI.^47^ As TFs play a key role in shaping the transcriptome, motif enrichment analysis in scATAC-seq data of mouse carotid artery in different flow conditions identified KLF2/KLF4 motifs in stable flow, while motifs for RELA, AP1, STAT1, and TEAD1 were enriched in accessible regions from disturbed flow conditions.^48^ Approaches developed for TF target and/or regulon-based analysis of scRNA-seq data^109,110^ revealed the possible role of FLI1 and TEAD1 in tumour ECs,^55^ and of SOX18 in human PAH.^51^

### 5.3 Contribution of the microenvironment to EC heterogeneity in disease

ECs plastically adapt to the physiological needs of different tissues. Unsurprisingly therefore, signals in the microenvironment shape the EC subtype landscape.^111^ ECs acquire a specialized role depending on their location and status in physiological conditions that can make them more or less responsive to certain stimuli in disease. For instance, in cerebral cavernous malformation, venous capillary ECs are the main contributor of the lesion, as arterial ECs remain non-responsive to the transformation.^97^ Furthermore, HEVs in lymph nodes possess an activated phenotype that is lost upon changes to the microenvironment such as inhibition of lymphotoxin-β receptor signalling.^104^

Complex communicative circuits between ECs and other cell types play a key role in disease pathogenesis (*Figure 3*). For example, tumour aggressiveness is regulated through a crosstalk of ECs with cancer cells or tumour-associated macrophages in the microenvironment, regulating (among others) induction of metastasis and tumour angiogenesis.^112,113^ Moreover, interactions between ECs and cardiomyocytes are key during development and cardiac homeostasis, and become dysregulated in cardiovascular disease.^114,115^

Cell–cell communication and interaction can be assessed in scRNA-seq data by an unbiased analysis of receptor–ligand interaction (RLI) pairs using popular tools such as CellPhoneDB^116^ or more recent and comprehensive tools including CellChat^117^ and NicheNet,^118^ detailed hereafter in the ‘Recent Advances & Future Perspectives’ section of this review. Increased interactions of ECs with other cells were detected in the heart of postnatal Day 8 mice 3 days after MI^47^ but also in human atherosclerotic plaques.^49^ In the murine regenerative heart, R-Spondin was identified as an EC ligand expressed by epithelial cells with a pro-angiogenic effect to ECs *in vitro*.^47^ ECs appear to receive communication from fibroblasts in the murine hyperoxic lung, with the ligand and receptor *Bmp5* and *Bmpr2* expressed by fibroblast and ECs, respectively.^87^ In atherosclerosis, the PDGF/PDGFRB interaction between myeloid cells and ECs led to the hypothesis of a myeloid-driven angiogenic contribution to plaque destabilization.^49^ In the heart, evidence of communication between fibroblasts and ECs was detected in both healthy and injured conditions using scRNA-seq and the proximity between fibroblasts and ECs was confirmed by immunofluorescence.^119^ As mentioned previously, a study of the murine ageing heart revealed the deterioration of this paracrine crosstalk, with *in vitro* experiments showing a reduced angiogenic property of the conditioned medium from heart-derived aged fibroblasts.^73^ In contrast, ECs might communicate with mesenchymal cells in human cirrhotic liver, where the scar-associated ECs express the non-canonical Notch ligand *JAG1, JAG2*, and *DLL4*, whereas the *NOTCH3* receptor is expressed by scar-associated mesenchymal cells.^59^ Co-culture experiments, using primary human hepatic stellate cells (HPCs) and ECs from cirrhotic livers, validated that this interaction promotes fibrillar collagen production by HPCs, which could be inhibited by perturbation of *NOTCH3* expression,^59^ highlighting the translational potential of findings identified through scRNA-seq and interactome analyses.

RLI analysis also highlighted cell–cell interactions in physiological conditions, with potential implication for development and disease. In the lung, the epithelium was identified as a key hub for spatially-restricted regulation of EC morphogenesis, by means of their preferential expression of semaphorins and VEGF family members, a phenomenon that is conserved across multiple species.^66^ Lastly, and in line with their well-appreciated immunoregulatory role, interactome analyses revealed novel interactions between pulmonary ECs and immune cells, including possible recruitment of *CX3CR1+* non-classical monocytes to ECs (*CX3CL1*+), and attraction of *CCR1+* dendritic cells to veins (*CCL23+*), bronchial vessels (*CCL14+*), and lymphocytes (*CCL5+*),^69^ highlighting interesting avenues for future research in light of lung cancer and/or inflammatory disease.

Overall, a high level of EC heterogeneity has been observed across developmental, physiological and pathological conditions. Further investigation into this heterogeneity may help understand therapy resistance mechanisms and should be factored into future EC-focused therapeutic development.

## 6. Therapeutic implications

### 6.1 Anti-angiogenic therapies in cancer-targets and resistance

**Figure 4:**
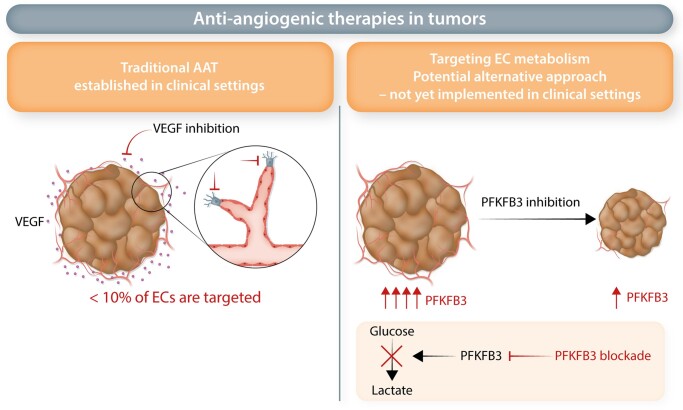
Anti-angiogenic therapies in tumours. *Traditional AAT*: Traditional AAT therapies target angiogenic growth factors, such as VEGF. VEGF-inhibition leads to inhibition of <10% of all ECs (including tip cells). *Targeting EC metabolism*: A potential alternative approach to inhibit angiogenesis in tumourigenesis presents targeting EC metabolism. Here inhibition of the glycolytic activator PFKFB3 has led to decreased tumour angiogenesis and impaired tumour growth in animal models. However, unlike traditional AAT, this approach has not yet been established in the clinical setting.

As angiogenesis is critical for a variety of diseases, therapies have been devised to either promote or inhibit angiogenesis.^120^ While pro-angiogenic efforts promise to offer novel therapeutic opportunities for cardiovascular disease and diabetes, here we focus on anti-angiogenic therapies (AATs). Cancer presents one of the main pathologies for which AAT is used, due to the critical role of angiogenesis in cancer progression and metastasis.^121^ Currently approved AATs centre around blocking the key pro-angiogenic target VEGF, though other targets are emerging (*Figure 4*). While initially designed to prune the tumour vasculature,^122–124^ current clinical trials explore whether VEGF-blockade can improve immunotherapy by normalizing the tumour vasculature.^125^ The success of VEGF-blockade therapy is however tampered by insufficient efficacy and resistance.^126,127^ Several resistance mechanisms have been proposed, ranging from alternative growth factor signalling to other modes of tumour vascularization, such as vessel co-option,^128,129^ but only recent studies explored additional mechanisms at the single EC level.^53,130^

In a mouse lung cancer model, tip cells and breach cells (putatively assisting tip cells to lead the vessel sprout^53^) represent the EC subtypes most sensitive to VEGF blockade,^53^ whereas other EC subtypes were less or differentially sensitive. In fact, post-capillary vein ECs increased in abundance upon anti-VEGF treatment.^53^ Whether the increases in capillary and post-capillary vein ECs is a consequence of switching from SA to vessel co-option (a known escape mechanism to AAT therapy^131^) remains to be determined. This may explain, at least in part, the limited success and therapeutic immunity towards AAT.

In addition, the various distinct EC types identified by single-cell transcriptomic studies might also contribute to a better understanding of AAT resistance.^53,132^ Tip cells, which are the presumed key targets of AAT, amount to fewer than 10% of all ECs within lung tumours,^53^ thus the majority of ECs is in fact not targeted by AAT (*Figure 4*). Differences in the composition of different EC subtypes in tumours from distinct patients^53^ might furthermore explain why some patients respond better than others to AAT. Moreover, venous ECs in tumours contain a subset of so-called resident endothelial stem cells (rESCs).^53^ rESCs were also identified in large vessels of multiple murine organs and showed self-renewal capacity as well as contributed to vessel regeneration in different models of vessel injury.^65,92,93^ As venous ECs expand upon AAT,^53^ it raises the question whether these rESCs might reconstitute vessels upon AAT, thereby contributing to therapy resistance. Endothelial progenitor cells were identified in human metastatic lung adenocarcinoma.^133^ Moreover, aldehyde dehydrogenase (ALDH)-positive ECs with stem-like properties were found in melanoma (xenograft models) and human renal cell carcinomas. These ALDH-positive stem-like ECs display pro-angiogenic properties, and resisted to chemotherapy treatment.^134,135^ How such progenitor-like ECs are impacted by AAT remains to be determined. Future studies will determine whether such cells are present in other tumour types, and contribute to AAT resistance by induction of neoangiogenesis upon treatment. Interestingly, ‘Myc targets’ was amongst the top up-regulated pathways in tumour ECs in a single-cell analysis of human non-small cell lung cancer (NSCLC).^55^ Myc has been identified as a driver of the endothelial regeneration process,^93^ thereby raising the question whether progenitor-like ECs might arise in tumours, and if so, whether they harbour additional heterogeneity in terms of their transcriptome or their response to anti-cancer therapy/AAT. Of note, while several scRNA-seq studies identified EC populations with stem- or progenitor-like potential, future studies are needed to carefully assess potentially distinct vascular progenitors, which might be tissue and/or disease specific. Thus far, there is not yet a consensus definition of EC stem- and/or progenitor cells available based on scRNA-seq.

Alternative mechanisms of blood vessel growth, in addition to SA, which is the most studied form of angiogenesis, also need to be considered in the context of EC heterogeneity and its impact on cancer progression and therapy response. In fact, VEGF inhibition can induce substitute mechanisms of vessel growth, such as intussusceptive angiogenesis (IA)^136^ and vessel co-option.^131^ Also, vascular mimicry and vasculogenesis were identified as potential alternate processes that promote AAT resistance.^137,138^ However, single-cell studies investigating phenotypical and functional EC heterogeneity in these processes remain elusive. Such studies would be critical to identify novel targets to enable the control of pathologic angiogenesis by simultaneously attacking several aspects of vessel growth.

Importantly, the combination of AAT with other anti-cancer therapies, such as chemotherapy or immunotherapy has shown promising results not only in pre-clinical models but also in the clinic. In fact, several AAT agents (e.g. bevacizumab, aflibercept, sorafenib, sunitinib), apart from being approved as single-agent therapy, have reached approval in combination with chemotherapy, or as second-line therapy after patients progressed on chemotherapy.^139^ Moreover, the combination of interferon-alpha (IFN-α) treatment with anti-VEGF therapy has been approved by the FDA for treatment of metastatic renal cell carcinoma.^140^ With the advent of novel immunotherapies, such as immune checkpoint blockade, there are many new promising anti-cancer therapeutic opportunities.^139^ New insights into distinct EC phenotypes could help to develop more precise treatments tailored to target specific EC populations, which might create a favourable environment, in particular for immunotherapy to work. IMECs or other specialized EC phenotypes might offer such opportunities. For instance, HEVs are involved in the recruitment of different immune cells,^141^ thus promoting HEV growth is expected to be beneficial for enhancing the anti-cancer effect of immunotherapy. This concept to ‘tune rather than only prune’ is a novel strategy for future AAT.

### 6.2 EC metabolism as alternative target to modulate angiogenesis

More than a decade ago, ECs were shown to undergo metabolic changes to execute their various functions. This metabolic reprogramming is driven in part by different signalling cascades, for instance growth factor signalling (e.g. VEGF can induce glycolysis) or Notch signalling (Notch suppresses glycolysis in stalk cells).^142^ However, it is now clear that EC metabolism is not only necessary but also sufficient (independent of growth factors or other stimuli) to control EC function.^143^ Several metabolic pathways have been implicated in distinct functions. Single-cell studies alongside metabolomic investigations have uncovered several metabolically distinct EC subtypes. For instance, during SA, tip cells up-regulate glycolysis and amino acid metabolism to support migration.^142–144^ These metabolic pathways are also used by stalk cells (however, at lower levels), where they support proliferation and biomass production.^143^ Stalk cells as well as phalanx cells also rely on fatty acid oxidation (FAO).^145^ In quiescent phalanx ECs, FAO contributes to maintainence of their quiescent phenotype^146^ (*Figure 4*). It has also been recognized that different EC subsets display distinct metabolic signatures, in a tissue-specific manner.^41^ For instance, different metabolic transporters are most highly expressed in brain ECs, spleen ECs are enriched in cholesterol metabolism, while cardiac and muscle ECs show elevated fatty acid metabolism.^41^ For a detailed review of EC metabolism, and metabolic heterogeneity in different EC types, we refer to recent excellent reviews.^143,147,148^

When comparing ECs from healthy tissues to those in disease, different metabolic gene signatures were observed as well. For instance, compared to their respective controls, ECs from choroidal neovascularization or murine lung tumour models displayed an increase in gene expression related to several metabolic pathways, such as glycolysis, tricarboxylic acid cycle, oxidative phosphorylation (OXPHOS), one-carbon metabolism, and nucleotide synthesis.^65^ In line with these findings, single-cell analysis of colorectal, lung and ovarian cancer revealed that tip ECs in all three cancer types up-regulate glycolysis and OXPHOS gene signatures.^56^ Moreover, EC subtypes in human lung cancer also presented with metabolic gene adaptations compared to their healthy counterparts, with an up-regulation of genes involved in lipid metabolism in capillary tumour ECs, and increased prostaglandin metabolism in venous tumour ECs.^53^ Compared to ECs from early stage ground glass nodules adenocarcinoma, ECs from late stage solid lung adenocarcinoma were also enriched in metabolic gene processes,^149^ and circulating ECs from metastatic prostate cancer patients showed enriched metabolic gene expression compared to circulating ECs from healthy controls.^150^

The findings of EC metabolism as critical propeller to EC function, along with the observed metabolic changes in tumour ECs, led to the hypothesis that metabolic targeting of ECs might offer new therapeutic opportunities to keep tumour angiogenesis at bay (*Figure 4*). The glycolytic enzyme PFKFB3 regulates tip and stalk cell phenotypes, and associates with actin remodelling.^142^ Genetic silencing of PFKFB3 inhibited tip cell function and resulted in acquisition of a quiescent phenotype.^142^ Pharmacological inhibition of PFKFB3 with the inhibitor 3PO (3-(3-pyridinyl)-1-(4-pyridinyl)-2-propen-1-one) impeded vessel sprouting in models of retinal angiogenesis and vascular development in zebrafish.^144^ Notably, pathological angiogenesis in different disease models (age-related macular degeneration, retinopathy of prematurity, skin psoriasis, inflammatory bowel disease, and cancer) was also suppressed by 3PO treatment^144,151^ (*Figure 4*). Importantly, while pharmacological PFKFB3 inhibition impedes angiogenesis in pre-clinical models, the efficacy of the treatment in clinical settings remains to be tested (*Figure 4*). Moreover, blocking of FAO hampers pathological angiogenesis. Etomoxir, which inhibits the FAO enzyme Carnitine Palmitoyltransferase 1A (CPT1A) reduces pathological angiogenesis in a model of retinopathy of prematurity.^145^ Tip and stalk cells also rely on fatty acid synthesis.^143^ In fact, pharmacological inhibition of the fatty acid synthase (*FASN*) using Orlistat, reduces EC proliferation and angiogenesis in pathological ocular neovascularization and melanoma animal models.^152,153^ Thus far, no apparent off-target effects were discovered in preclinical models; however, it is critical to note that targeting metabolic pathways affects not specifically ECs, but all cell types. Therefore, the suitability of metabolic targets to specifically inhibit EC functions in patients remains to be investigated. However, as discussed in the following paragraph, recent developments in precision medicine might allow targeting of EC-specific metabolic pathways. In summary, these promising results demonstrate the need for future studies on the metabolic heterogeneity of ECs to identify additional metabolic targets.

### 6.3 Novel targets from single-cell studies—prioritization and targeting

**Figure 5:**
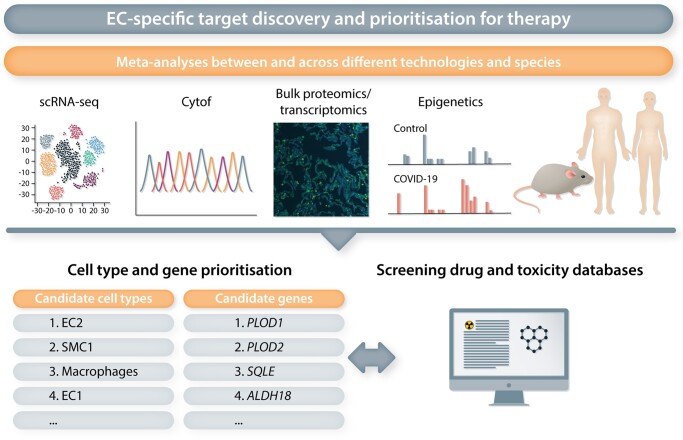
EC-specific target discovery and prioritization for therapy. Meta-analyses using different platforms (for instance scRNAseq, Cytof, Bulk proteomics/transcriptomics, epigenetic analyses etc.) and comparing data between different species (mouse, rat, human…) can narrow down candidate cell types and genes with important biological functions in a pathological setting. This approach focuses on genes/proteins repetitively up- or down-regulated in the pathological setting independent of the method used and congruently changed between different species. Drug and toxicity databases can then be exploited to identify potential drugs/drug classes to reverse the determined genes/gene signatures. The availability of FDA/EMA-approved drugs potentially capable of targeting certain genes can also help in target prioritization.

Whilst the unravelling of EC heterogeneity at single-cell resolution has led to the discovery of exciting novel and specialized EC subtypes with a presumable key role in disease, the prioritization of functionally important candidate (metabolic) genes that are most reflective of these EC subtypes remains a formidable challenge. It demands the development of efficient means to transcend the atlas-like descriptive listing of EC-subtype specific marker genes into the most promising functionally relevant and therapeutically targetable candidates, and various *in silico* methods have been developed and reported in the recent years to aid in this challenge. For instance, the use of an integrated (meta-)analysis of candidate gene expression across species, diseases and models identified *PLOD1* and *PLOD2* as novel angiogenic candidates.^53^ Silencing or inhibition of both genes furthermore impaired *in vitro* and *in vivo* vessel sprouting, validating the therapeutic potential of these genes.^53^ Moreover, a similar meta-analysis approach, yet combined with scRNA-seq data-tailored genome-scale metabolic models (GEMs), proved an efficient method for prioritization of *SQLE* and *ALDH18* as promising new metabolic targets for AAT^65^ (*Figure 5*). Again, *in vitro* and *in vivo* perturbation experiments confirmed the functional relevance of both genes for angiogenesis, stressing their translational potential.^65^

Querying of cell types enriched for trait-relevant genes based on genome-wide association studies (GWAS)^154,155^ represents another intriguing strategy for the identification of EC-specific genes associated with a particular disease or condition. For instance, a GWAS-based analysis of genes associated with cardiovascular disease was performed in a scRNA-seq study of human atherosclerotic plaques.^49^ Eight of such genes (*SHE, KCNN3, VAMP5, SEMA3F, HDAC9, GIMAP1, NOS3*, and *DOCK6*) showed an EC-enriched expression pattern, supporting EC contribution to the disease and providing crucial information for future functional characterizations.^49^ Furthermore, in scRNA-seq data of two rat models of PAH, relevance to the human disease was investigated by analysing the expression of genes implicated in PAH based on DisGeNET and the Comparative Toxicogenomics Database,^156^ and in a human PAH scRNA-seq study, differential expression of genes associated with hereditary PAH (e.g. *BMPR2, ENG, SMAD9*) was confirmed in several cell types, including ECs.^51^ Interestingly, the rat PAH scRNA-seq study also assessed the therapeutic potential of existing drugs in PAH, by means of *in silico* drug screening.^156^ This screening relied on the ‘Connectivity Map’ resource, which allows the comparison of scRNA-seq transcriptional signatures with a reference collection of drug-induced gene expression profiles from cultured human cells^157^ (*Figure 5*). Another recent method, Augur, allows prioritization of cellular subtypes most responsive to a biological perturbation,^158^ in lieu of the traditional prioritization based on differential gene expression. This enables the identification of the individual contributions of distinct cell types to a condition or their discrete responses to different treatments, thereby deciphering the roles of distinct cell subtypes on a broader scale.^158^ The *in silico* construction of multicellular disease models (MCDMs)^159^ is yet an additional method for target prioritization. This systems-level approach uses scRNA-seq data to construct models of disease-associated cell types, their expression profiles, and predicted cell-cell interactions. By integrating this method with disease context-specific genetic and epigenetic data, the possibility of identifying the most (therapeutically) relevant cell types was showcased in single-cell datasets of human and mouse rheumatoid arthritis.^159^ These novel approaches all showcased the ability of cell type and target prioritization from complex scRNA-seq datasets, and their application to EC-specific OMICs data promises to unveil important insights into vascular subtypes and marker genes most relevant for follow-up in a disease or condition-specific context (*Figure 5*).

While identifying the EC subtype and associated marker(s) most likely to be of therapeutic interest already poses a challenge, subsequent specific targeting of the prioritized vascular subset may present an even bigger hurdle. Developments in the selective targeting of an EC subtype, recently coined ‘precision angioscience’,^160^ will therefore be instrumental in translating EC-derived scRNA-seq data into clinically interesting and feasible follow-up studies. Selective delivery of small interfering RNAs (siRNAs), single-guide RNAs (sgRNAs), messenger RNAs (mRNAs), small molecules, and therapeutic proteins represents another strategy for specific targeting of the endothelium, and has thus far been experimentally achieved through the use of targeting ligands (for instance monoclonal antibodies), directed against EC-specific adhesion molecules or other surface markers. Vascular cell adhesion molecule-1 (VCAM1)-targeted nanoparticles have shown promising results in light of imaging inflamed or ischaemic tissues in the mouse.^161–164^ Furthermore, enzyme-antibody conjugates and nanoparticle formulation aimed at specific targeting of the pulmonary^165,166^ or splenic^167^ murine vasculature have been reported so far, often with negligible alterations in non-vascular cell types or other tissues.

Although promising, *in vivo* gene delivery to a particular EC subtype identified by scRNA-seq has thus far not been achieved but may harbour benefits over pan-EC targeted strategies in terms of toxicity to other parts of the vascular bed within and outside of the tissue of interest. One major reason why targeting of specific EC subtypes identified by scRNA-seq studies has not yet been achieved, is the lack of consensus marker genes for distinct EC subpopulations. Future scRNA-seq analyses might provide further insights into construction of specific promoters for inclusion into gene therapy vectors in order to selectively target specific EC populations. This strategy however depends on the mutual exclusivity of EC subtype specific marker genes, and may be more challenging in case of tissues, where EC expression signatures exhibit spatial zonation, as for instance shown in the hepatic vasculature.^168^

## 7. Recent advances and future perspectives

### 7.1 A compendium of all publicly available single ECs

Despite the vast amount of scRNA-seq studies published to date, the abundance of the vascular compartment within individual studies is often relatively low, precluding a detailed and all-encompassing interrogation of its heterogeneity. Increasing the magnitude of EC-derived single-cell datasets, by performing a joint analysis across all publicly available studies, could offer a solution to this problem. Although seemingly straightforward, this strategy nevertheless faces multiple challenges, including the need for effective batch effect correction, lack of standardization in EC isolation protocols, and variation in single-cell data analysis, subclustering and annotation strategies (see *Box 3*).

Box 3 Challenges of integrating multiple single EC datasets An integrated analysis of ECs extracted from multiple, publicly available single-cell datasets would provide a solution to the problem of overall low numbers of high-quality ECs in most individual (whole tissue) studies. However, this strategy faces multiple challenges:

- Unavoidable ‘batches’ across single-cell datasets arise when they are generated in different labs, and/or comprise different experimental models, sample cohorts, library preparation methods, or sequencing platforms. If not properly accounted for, these batch effects could severely bias conclusions drawn from comparative and/or integrated analyses. Despite the rapid development, optimization, and benchmarking of user-friendly data integration or batch correction methods for single-cell datasets,^211–214^ their use is limited to only certain aspects of downstream data analyses, and finding a proper balance between aligning multiple datasets while preserving key biological variation remains challenging. Not surprisingly, batch correction is recognized as one of the major challenges in the single-cell OMICs community.^215^
- With the increasing number of published single-cell studies, insufficient standardization of tissue isolation, as well as inconsistencies in annotation of EC subtypes are arising as a major hurdle in the vascular single-cell field. Usage of different isolation protocols inevitably leads to variation in the overall yield of cellular lineages, and the vascular compartment is no exception.^216^ Standardized protocols for EC isolation from various mouse tissues are rising,^217–219^ and optimized pipelines for pan-cell type isolation of single cells or nuclei from human tumour samples are also being developed.^216^ The continuation of such developments in additional tissues, conditions and species are expected to reduce discrepancies in overall EC/EC subtype yields across studies.
- The categorization of ECs into transcriptomically distinct phenotypes or subgroups within the identified vascular compartments, which by itself is not a trivial pursuit, varies substantially across studies. Whereas this variability can likely be attributed to differences in the overall EC yield across these studies (indeed, studies analysing enriched EC populations generally report a higher number of transcriptomically distinct EC subtypes as compared to whole-tissue analyses^41,53,55,65,133^), differences in the applied subclustering parameters and annotation strategies may also play a role.

The latter issue is expected to improve in the coming years with the advancement of automated cell type annotation tools, which are rising in number and user-friendliness,^169–172^ but even more so with the development of tools like Azimuth,^173^ providing rapid and automated mapping, visualization and annotation of single-cell datasets through an online web application. Yet, these tools often provide reference datasets representing major cellular lineages in various tissues/organs but preclude annotation of different EC subtypes within a particular tissue or vascular bed. There is thus a need for the generation of tissue-specific ‘gold standard’ vascular atlases, to both improve and progress standardization of EC OMICs annotations. A recent integration of 6 lung scRNA-seq datasets resulted in joint profiling of over 15 000 ECs from 73 individuals,^174^ and although not covering the full spectrum of published (healthy/normal) lung single EC RNA-seq data, this study provided one of the first in-depth reference atlases of healthy/normal lung ECs and is likely to aid annotation of future pulmonary EC studies in health and disease. When such efforts will be combined with automated cell type mapping tools and standardized whole tissue/EC isolation protocols, harmonized EC annotation across laboratories, tissues and experimental setups should be feasible in the foreseeable future.

Another obstacle in integrated analysis of EC OMICs data is represented by the inconsistent formats in which raw data is deposited, and the (sometimes) severe lack of detail regarding sample origin information and data processing parameters. The availability of processed counts and annotated metadata is furthermore limited, yet inevitable to ensure reproducibility of the data across labs of different expertise. Data-sharing methods also become increasingly variable, complicating uniform methods of dataset curation. While lab-hosted servers, offering virtual exploration and downloading of data, are rising in popularity and enable non-bioinformatics focused labs an affordable and reliable method of data exploration, a more centralized storage platform would greatly enhance our ability to study vascular OMICs in a streamlined and comprehensive manner. Various recent efforts aimed at offering solace, either by generation of free-of-charge portals harbouring curated and harmonized processed datasets, or frequently updated overviews of published scRNA-seq datasets.^175–178^ Specialized databases, like *JingleBells*^179^ for immune cells, *cancerSEA*^180^ for cancer cell states, *The Human Cell Atlas* portal for all tissues and cell types of the human body,^181^ or the *NIH Human Biomolecular Atlas Program (HuBMAP*),^182^ furthermore provide tempting field-specific opportunities in terms of scRNA-seq data exploration and analysis. Yet, none of these portals/efforts capture the complete spectrum of published datasets, and their usefulness relies on continuous data curation and updates.

**Figure 6:**
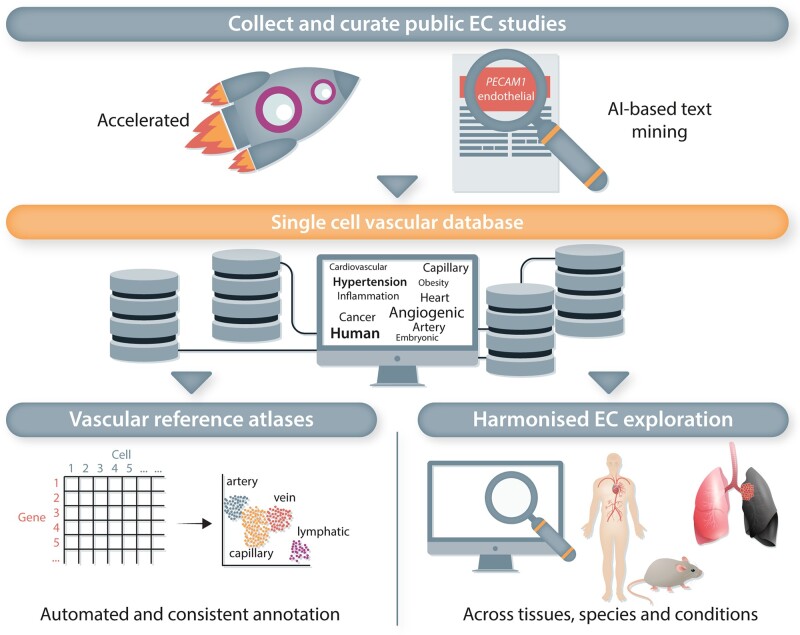
A single-cell vascular database. Single-cell OMICs studies generate vast amounts of data. The challenge is to identify biologically relevant EC phenotypes and disease-specific changes in ECs. Here, text-mining tools can be tailored to identify OMICs publications including ECs, to aid in the generation of an all-encompassing repository of EC OMICs data. Such a database will facilitate automated and consistent EC annotation, as well as the comparison of ECs between different tissues, species and conditions, advancing and harmonizing data-driven research in vascular biology.

If we are to make progress in deciphering vascular heterogeneity across species, tissues and conditions, a dedicated portal housing all publicly available vascular-centred single OMICs data appears to become a key milestone waiting to be accomplished. However, as the ever-increasing number of single-cell datasets published is becoming difficult to curate, a demand for artificial intelligence (AI)-based data-mining approaches is likely to arise in parallel to realize such an effort in an all-encompassing manner. Implementation of natural language processing strategies and recent developments in their specific moulding towards biomedical sciences appear promising.^183,184^ Amidst the current single-cell OMICs ‘tsunami’ of data, tailoring of text-mining tools towards identifying OMICs publications harbouring a particular cell type of interest (in this case, ECs) has the potential to greatly enhance their identification and prioritization, accelerating the generation of comprehensive single EC OMICs repositories and furthering data-driven research in the (vascular) biology field (*Figure 6*).

### 7.2 ECs never work alone-interactomes and spatial resolution

**Figure 7:**
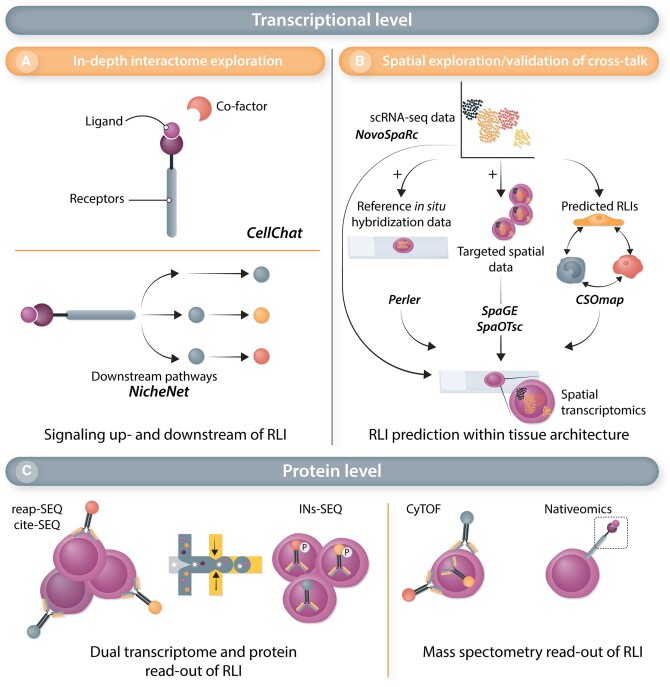
Extension and validation of RLIs predicted from single-cell data. An overview of methods for further exploration and validation of predicted RLIs from scRNA-seq data, either on the transcriptome level (upper panel) or protein level (lower panel). (*A*) Computational tools can be used to retrieve information regarding interactions between ligands, receptors and their co-factors (CellChat), or the intracellular response of cell types on the ‘receiving end’ of predicted RLIs (NicheNet). (*B*) RLIs can also be placed in a spatial context by implementation of computational tools allowing the integration of (i) scRNA-seq data with reference *in situ* hybridization data (Perler), or (ii) scRNA-seq data and its predicted RLI landscape (CSOmap). Spatial information can also be reconstructed *de novo* using scRNA-seq data (novoSpaRc), or by means of mapping untargeted scRNA-seq data to smaller, targeted spatial transcriptomics datasets (SpaGE, SpaOTsc). (*C*) Protein level exploration of RLIs can be achieved by applying established methods aimed at generating a dual transcriptome and protein read-out in scRNA-seq experiments (CITE-seq, REAP-seq, INs-seq), or by using mass-spectometry based methods [cytometry by time of flight (CyTOF), Nativeomics].

As described above, intricate cellular communication between ECs and their neighbouring cells are of vital importance for maintaining vascular homeostasis and remodelling, and recent advances in the development of interactome prediction tools for single-cell data revealed intriguing findings regarding the interplay of ECs and other cell types.^47,49,59,87^ Although fascinating, it must be noted that the findings and interactions resulting from RLI analysis represent predictions, requiring functional validation. Recently developed tools provide more comprehensive solutions, including CellChat,^117^ taking into account interactions between ligands, receptors and their co-factors, or NicheNet,^118^ aimed at diving deeper into the intracellular response of cell types on the ‘receiving end’ of these predicted interactions (*Figure 7A*). Ultimately, however, RLIs can be more accurately investigated when positional information is preserved. Advances in spatial transcriptomics, crowned as ‘Method of the year 2020’ by *Nature Methods*,^185^ presumably hold great promise for future enhancements in studying the interplay between ECs and their environment in a tissue architecture-dependent context (*Figure 7B*). Interestingly, several computational tools have been recently developed with the aim to provide a more cost-effective alternative to spatial transcriptomics, either by integration of scRNA-seq data with reference *in situ* hybridization data (Perler^186^) prediction of cellular coordinates in a three-dimensional pseudo-space based on input scRNA-seq data and known ligand-receptor interactions (CSOmap^187^) *de novo* spatial reconstruction of single-cell gene expression (novoSpaRc^188^) or prediction of whole-transcriptome expressions in their spatial configuration by mapping of untargeted scRNA-seq data to smaller, targeted spatial transcriptomics datasets (SpaGE,^189^ SpaOTsc^190^) (*Figure 7B*).

Finally, translation of cellular cross-talk predictions to the protein level, for instance by applying established methods including CITE-seq,^191^ REAP-seq,^192^ or cytometry by time of flight (CyTOF),^193^ or the more newly developed Nativeomics^194^ (allowing detection of intact ligand–receptor assemblies using mass spectrometry), INs-seq^195^ (allowing more accurate exploration of TFs, active signalling networks and metabolic activity by parallel transcriptome and intracellular proteomic profiling at single-cell resolution), or single-cell proteomics^196^ will be essential to complement and finetune EC-interactomes predicted from scRNA-seq data (*Figure 7C*). Lastly, as spatial juxtaposition of an EC and another cell type does not automatically imply their active communication, the abovementioned tools will undoubtedly help prioritize the interactions that are most promising for further functional validation.

## 8. Conclusion

Collectively, EC OMICs studies have opened up a staggering amount of data readily available for analysis, of which we have currently only scratched the surface. Nevertheless, the single endothelial landscape uncovered thus far has revealed an intriguing degree of transcriptional heterogeneity and has already propelled the vascular biology field at unprecedented speed. Further efforts aimed at unravelling the associated biological and functional relevance of this heterogeneity will undoubtedly help forward our understanding of the molecular drivers by leaps and bounds, and reveal the translational potential of exploiting EC heterogeneity for the development of novel AAT or endothelial-targeted therapies.

## Authors’ contributions

L.M.B., S.-H.C., J.R., and L.P.M.H.d.R. wrote the manuscript, and designed the figures. A.H.B. and P.C. conceptualized the manuscript. All authors approved the manuscript.

## Data Availability

There are no new data associated with this article.

## References

[1] Deanfield JE , Halcox JP, Rabelink TJ. Endothelial function and dysfunction: testing and clinical relevance. Circulation 2007;115:1285–1295. 17353456 10.1161/CIRCULATIONAHA.106.652859

[2] Tesfamariam B , DeFelice AF. Endothelial injury in the initiation and progression of vascular disorders. Vascul Pharmacol 2007;46:229–237. 17218160 10.1016/j.vph.2006.11.005

[3] Rajendran P , Rengarajan T, Thangavel J, Nishigaki Y, Sakthisekaran D, Sethi G, Nishigaki I. The vascular endothelium and human diseases. Int J Biol Sci 2013;9:1057–1069. 24250251 10.7150/ijbs.7502 PMC3831119

[4] Zenaro E , Piacentino G, Constantin G. The blood-brain barrier in Alzheimer's disease. Neurobiol Dis 2017;107:41–56. 27425887 10.1016/j.nbd.2016.07.007 PMC5600438

[5] Altschuler SJ , Wu LF. Cellular heterogeneity: do differences make a difference? Cell 2010;141:559–563. 20478246 10.1016/j.cell.2010.04.033 PMC2918286

[6] Aird WC. Endothelial cell heterogeneity. Cold Spring Harb Perspect Med 2012;2:a006429. 22315715 10.1101/cshperspect.a006429 PMC3253027

[7] Aird WC. Phenotypic heterogeneity of the endothelium: I. Structure, function, and mechanisms. Circ Res 2007;100:158–173. 17272818 10.1161/01.RES.0000255691.76142.4a

[8] Aird WC. Phenotypic heterogeneity of the endothelium: II. Representative vascular beds. Circ Res 2007;100:174–190. 17272819 10.1161/01.RES.0000255690.03436.ae

[9] Goldman SL , MacKay M, Afshinnekoo E, Melnick AM, Wu S, Mason CE. The impact of heterogeneity on single-cell sequencing. Front Genet 2019;10:8. 30881372 10.3389/fgene.2019.00008 PMC6405636

[10] Ruoslahti E , Rajotte D. An address system in the vasculature of normal tissues and tumors. Annu Rev Immunol 2000;18:813–827. 10837076 10.1146/annurev.immunol.18.1.813

[11] Simonson AB , Schnitzer JE. Vascular proteomic mapping *in vivo*. J Thromb Haemost 2007;5(Suppl. 1):183–187. 17635725 10.1111/j.1538-7836.2007.02551.x

[12] Griffin NM , Schnitzer JE.Chapter8. Proteomic mapping of the vascular endothelium *in vivo* for vascular targeting. Methods Enzymol 2008;445:177–208. 19022060 10.1016/S0076-6879(08)03008-5

[13] Stamper HB Jr , Woodruff JJ. Lymphocyte homing into lymph nodes: *in vitro* demonstration of the selective affinity of recirculating lymphocytes for high-endothelial venules. J Exp Med 1976;144:828–833. 956727 10.1084/jem.144.3.828 PMC2190398

[14] Hill TA , Stanford MR, Graham EM, Dumonde DC, Brown KA. A new method for studying the selective adherence of blood lymphocytes to the microvasculature of human retina. Invest Ophthalmol Vis Sci 1997;38:2608–2618. 9375580

[15] Grober JS , Bowen BL, Ebling H, Athey B, Thompson CB, Fox DA, Stoolman LM. Monocyte-endothelial adhesion in chronic rheumatoid arthritis. *In situ* detection of selectin and integrin-dependent interactions. J Clin Invest 1993;91:2609–2619. 7685772 10.1172/JCI116500 PMC443325

[16] Gallatin WM , Weissman IL, Butcher EC. A cell-surface molecule involved in organ-specific homing of lymphocytes. Nature 1983;304:30–34. 6866086 10.1038/304030a0

[17] Pasqualini R , Ruoslahti E. Organ targeting *in vivo* using phage display peptide libraries. Nature 1996;380:364–366. 8598934 10.1038/380364a0

[18] Arap W , Haedicke W, Bernasconi M, Kain R, Rajotte D, Krajewski S, Ellerby HM, Bredesen DE, Pasqualini R, Ruoslahti E. Targeting the prostate for destruction through a vascular address. Proc Natl Acad Sci USA 2002;99:1527–1531. 11830668 10.1073/pnas.241655998 PMC122224

[19] Sengoelge G , Luo W, Fine D, Perschl AM, Fierlbeck W, Haririan A, Sorensson J, Rehman T-U, Hauser P, Trevick JS, Kulak SC, Wegner B, Ballermann BJ. A SAGE-based comparison between glomerular and aortic endothelial cells. Am J Physiol Renal Physiol 2005;288:F1290–F1300. 15657302 10.1152/ajprenal.00076.2004

[20] Sengoelge G , Winnicki W, Kupczok A, von Haeseler A, Schuster M, Pfaller W, Jennings P, Weltermann A, Blake S, Sunder-Plassmann G. A SAGE based approach to human glomerular endothelium: defining the transcriptome, finding a novel molecule and highlighting endothelial diversity. BMC Genomics 2014;15:725. 25163811 10.1186/1471-2164-15-725 PMC4156628

[21] Chi J-T , Chang HY, Haraldsen G, Jahnsen FL, Troyanskaya OG, Chang DS, Wang Z, Rockson SG, van de Rijn M, Botstein D, Brown PO. Endothelial cell diversity revealed by global expression profiling. Proc Natl Acad Sci USA 2003;100:10623–10628. 12963823 10.1073/pnas.1434429100 PMC196854

[22] Schweighofer B , Rohringer S, Proll J, Holnthoner W. A microarray analysis of two distinct lymphatic endothelial cell populations. Genom Data 2015;4:115–118. 26484194 10.1016/j.gdata.2015.04.005 PMC4535941

[23] Chen BP , Li YS, Zhao Y, Chen KD, Li S, Lao J, Yuan S, Shyy JY, Chien S. DNA microarray analysis of gene expression in endothelial cells in response to 24-h shear stress. Physiol Genomics 2001;7:55–63. 11595792 10.1152/physiolgenomics.2001.7.1.55

[24] Voyta JC , Via DP, Butterfield CE, Zetter BR. Identification and isolation of endothelial cells based on their increased uptake of acetylated-low density lipoprotein. J Cell Biol 1984;99:2034–2040. 6501412 10.1083/jcb.99.6.2034 PMC2113570

[25] Nolan DJ , Ginsberg M, Israely E, Palikuqi B, Poulos MG, James D, Ding B-S, Schachterle W, Liu Y, Rosenwaks Z, Butler JM, Xiang J, Rafii A, Shido K, Rabbany SY, Elemento O, Rafii S. Molecular signatures of tissue-specific microvascular endothelial cell heterogeneity in organ maintenance and regeneration. Dev Cell 2013;26:204–219. 23871589 10.1016/j.devcel.2013.06.017 PMC3873200

[26] Fina L , Molgaard HV, Robertson D, Bradley NJ, Monaghan P, Delia D, Sutherland DR, Baker MA, Greaves MF. Expression of the CD34 gene in vascular endothelial cells. Blood 1990;75:2417–2426. 1693532

[27] Ghitescu LD , Crine P, Jacobson BS. Antibodies specific to the plasma membrane of rat lung microvascular endothelium. Exp Cell Res 1997;232:47–55. 9141620 10.1006/excr.1997.3490

[28] Pusztaszeri MP , Seelentag W, Bosman FT. Immunohistochemical expression of endothelial markers CD31, CD34, von Willebrand factor, and Fli-1 in normal human tissues. J Histochem Cytochem 2006;54:385–395. 16234507 10.1369/jhc.4A6514.2005

[29] Ponder BA , Wilkinson MM. Organ-related differences in binding of Dolichos biflorus agglutinin to vascular endothelium. Dev Biol 1983;96:535–541. 6832482 10.1016/0012-1606(83)90191-4

[30] Belloni PN , Nicolson GL. Differential expression of cell surface glycoproteins on various organ-derived microvascular endothelia and endothelial cell cultures. J Cell Physiol 1988;136:398–410. 3170638 10.1002/jcp.1041360303

[31] McIntosh DP , Tan XY, Oh P, Schnitzer JE. Targeting endothelium and its dynamic caveolae for tissue-specific transcytosis *in vivo*: a pathway to overcome cell barriers to drug and gene delivery. Proc Natl Acad Sci USA 2002;99:1996–2001. 11854497 10.1073/pnas.251662398 PMC122308

[32] Arap W , Pasqualini R, Ruoslahti E. Cancer treatment by targeted drug delivery to tumor vasculature in a mouse model. Science 1998;279:377–380. 9430587 10.1126/science.279.5349.377

[33] Gunawardana H , Romero T, Yao N, Heidt S, Mulder A, Elashoff DA, Valenzuela NM. Tissue-specific endothelial cell heterogeneity contributes to unequal inflammatory responses. Sci Rep 2021;11:1949. 33479269 10.1038/s41598-020-80102-w PMC7820348

[34] Mincarelli L , Lister A, Lipscombe J, Macaulay IC. Defining cell identity with single-cell omics. Proteomics 2018;18:e1700312. 29644800 10.1002/pmic.201700312 PMC6175476

[35] Vanlandewijck M , He L, Mäe MA, Andrae J, Ando K, Del Gaudio F, Nahar K, Lebouvier T, Laviña B, Gouveia L, Sun Y, Raschperger E, Räsänen M, Zarb Y, Mochizuki N, Keller A, Lendahl U, Betsholtz C. A molecular atlas of cell types and zonation in the brain vasculature. Nature 2018;554:475–480. 29443965 10.1038/nature25739

[36] Karaiskos N , Rahmatollahi M, Boltengagen A, Liu H, Hoehne M, Rinschen M, Schermer B, Benzing T, Rajewsky N, Kocks C, Kann M, Müller R-U. A single-cell transcriptome atlas of the mouse glomerulus. J Am Soc Nephrol 2018;29:2060–2068. 29794128 10.1681/ASN.2018030238 PMC6065081

[37] Dumas SJ , Meta E, Borri M, Goveia J, Rohlenova K, Conchinha NV, Falkenberg K, Teuwen L-A, de Rooij L, Kalucka J, Chen R, Khan S, Taverna F, Lu W, Parys M, De Legher C, Vinckier S, Karakach TK, Schoonjans L, Lin L, Bolund L, Dewerchin M, Eelen G, Rabelink TJ, Li X, Luo Y, Carmeliet P. Single-cell RNA sequencing reveals renal endothelium heterogeneity and metabolic adaptation to water deprivation. J Am Soc Nephrol 2020;31:118–138. 31818909 10.1681/ASN.2019080832 PMC6935008

[38] MacParland SA , Liu JC, Ma X-Z, Innes BT, Bartczak AM, Gage BK, Manuel J, Khuu N, Echeverri J, Linares I, Gupta R, Cheng ML, Liu LY, Camat D, Chung SW, Seliga RK, Shao Z, Lee E, Ogawa S, Ogawa M, Wilson MD, Fish JE, Selzner M, Ghanekar A, Grant D, Greig P, Sapisochin G, Selzner N, Winegarden N, Adeyi O, Keller G, Bader GD, McGilvray ID. Single cell RNA sequencing of human liver reveals distinct intrahepatic macrophage populations. Nat Commun 2018;9:4383. 30348985 10.1038/s41467-018-06318-7 PMC6197289

[39] Aizarani N , Saviano A, Sagar Mailly L, Durand S, Herman JS, Pessaux P, Baumert TF, Grün D. A human liver cell atlas reveals heterogeneity and epithelial progenitors. Nature 2019;572:199–204. 31292543 10.1038/s41586-019-1373-2 PMC6687507

[40] Halpern KB , Shenhav R, Matcovitch-Natan O, Toth B, Lemze D, Golan M, Massasa EE, Baydatch S, Landen S, Moor AE, Brandis A, Giladi A, Avihail AS, David E, Amit I, Itzkovitz S. Single-cell spatial reconstruction reveals global division of labour in the mammalian liver. Nature 2017;542:352–356. 28166538 10.1038/nature21065 PMC5321580

[41] Kalucka J , de Rooij LPMH, Goveia J, Rohlenova K, Dumas SJ, Meta E, Conchinha NV, Taverna F, Teuwen L-A, Veys K, García-Caballero M, Khan S, Geldhof V, Sokol L, Chen R, Treps L, Borri M, de Zeeuw P, Dubois C, Karakach TK, Falkenberg KD, Parys M, Yin X, Vinckier S, Du Y, Fenton RA, Schoonjans L, Dewerchin M, Eelen G, Thienpont B, Lin L, Bolund L, Li X, Luo Y, Carmeliet P. Single-cell transcriptome atlas of murine endothelial cells. Cell 2020;180:764–779.e20. 32059779 10.1016/j.cell.2020.01.015

[42] Paik DT , Tian L, Williams IM, Rhee S, Zhang H, Liu C, Mishra R, Wu SM, Red-Horse K, Wu JC. Single-cell RNA sequencing unveils unique transcriptomic signatures of organ-specific endothelial cells. Circulation 2020;142:1848–1862. 32929989 10.1161/CIRCULATIONAHA.119.041433 PMC7658053

[43] Huang X , Shen W, Veizades S, Liang G, Sayed N, Nguyen PK. Single-cell transcriptional profiling reveals sex and age diversity of gene expression in mouse endothelial cells. Front Genet 2021;12:590377. 33679877 10.3389/fgene.2021.590377 PMC7929607

[44] Tabula Muris Consortium . A single-cell transcriptomic atlas characterizes ageing tissues in the mouse. Nature 2020;583:590–595. 32669714 10.1038/s41586-020-2496-1 PMC8240505

[45] Kashima Y , Sakamoto Y, Kaneko K, Seki M, Suzuki Y, Suzuki A. Single-cell sequencing techniques from individual to multiomics analyses. Exp Mol Med 2020;52:1419–1427. 32929221 10.1038/s12276-020-00499-2 PMC8080663

[46] Baran-Gale J , Chandra T, Kirschner K. Experimental design for single-cell RNA sequencing. Brief Funct Genomics 2018;17:233–239. 29126257 10.1093/bfgp/elx035 PMC6063265

[47] Wang Z , Cui M, Shah AM, Tan W, Liu N, Bassel-Duby R, Olson EN. Cell-type-specific gene regulatory networks underlying murine neonatal heart regeneration at single-cell resolution. Cell Rep 2020;33:108472. 33296652 10.1016/j.celrep.2020.108472 PMC7774872

[48] Andueza A , Kumar S, Kim J, Kang D-W, Mumme HL, Perez JI, Villa-Roel N, Jo H. Endothelial reprogramming by disturbed flow revealed by single-cell RNA and chromatin accessibility study. Cell Rep 2020;33:108491. 33326796 10.1016/j.celrep.2020.108491 PMC7801938

[49] Depuydt MAC , Prange KHM, Slenders L, Örd T, Elbersen D, Boltjes A, de Jager SCA, Asselbergs FW, de Borst GJ, Aavik E, Lönnberg T, Lutgens E, Glass CK, den Ruijter HM, Kaikkonen MU, Bot I, Slütter B, van der Laan SW, Yla-Herttuala S, Mokry M, Kuiper J, de Winther MPJ, Pasterkamp G. Microanatomy of the human atherosclerotic plaque by single-cell transcriptomics. Circ Res 2020;127:1437–1455. 32981416 10.1161/CIRCRESAHA.120.316770 PMC7641189

[50] Pijuan-Sala B , Griffiths JA, Guibentif C, Hiscock TW, Jawaid W, Calero-Nieto FJ, Mulas C, Ibarra-Soria X, Tyser RCV, Ho DLL, Reik W, Srinivas S, Simons BD, Nichols J, Marioni JC, Göttgens B. A single-cell molecular map of mouse gastrulation and early organogenesis. Nature 2019;566:490–495. 30787436 10.1038/s41586-019-0933-9 PMC6522369

[51] Saygin D , Tabib T, Bittar HET, Valenzi E, Sembrat J, Chan SY, Rojas M, Lafyatis R. Transcriptional profiling of lung cell populations in idiopathic pulmonary arterial hypertension. Pulm Circ 2020;10:1. 10.1177/2045894020908782 PMC7052475 32166015

[52] Adams TS , Schupp JC, Poli S, Ayaub EA, Neumark N, Ahangari F, Chu SG, Raby BA, DeIuliis G, Januszyk M, Duan Q, Arnett HA, Siddiqui A, Washko GR, Homer R, Yan X, Rosas IO, Kaminski N. Single-cell RNA-seq reveals ectopic and aberrant lung-resident cell populations in idiopathic pulmonary fibrosis. Sci Adv 2020;6:eaba1983. 32832599 10.1126/sciadv.aba1983 PMC7439502

[53] Goveia J , Rohlenova K, Taverna F, Treps L, Conradi L-C, Pircher A, Geldhof V, de Rooij LPMH, Kalucka J, Sokol L, García-Caballero M, Zheng Y, Qian J, Teuwen L-A, Khan S, Boeckx B, Wauters E, Decaluwé H, De Leyn P, Vansteenkiste J, Weynand B, Sagaert X, Verbeken E, Wolthuis A, Topal B, Everaerts W, Bohnenberger H, Emmert A, Panovska D, De Smet F, Staal FJT, Mclaughlin RJ, Impens F, Lagani V, Vinckier S, Mazzone M, Schoonjans L, Dewerchin M, Eelen G, Karakach TK, Yang H, Wang J, Bolund L, Lin L, Thienpont B, Li X, Lambrechts D, Luo Y, Carmeliet P. An integrated gene expression landscape profiling approach to identify lung tumor endothelial cell heterogeneity and angiogenic candidates. Cancer Cell 2020;37:21–36.e13. 31935371 10.1016/j.ccell.2019.12.001

[54] Habermann AC , Gutierrez AJ, Bui LT, Yahn SL, Winters NI, Calvi CL, Peter L, Chung M-I, Taylor CJ, Jetter C, Raju L, Roberson J, Ding G, Wood L, Sucre JMS, Richmond BW, Serezani AP, McDonnell WJ, Mallal SB, Bacchetta MJ, Loyd JE, Shaver CM, Ware LB, Bremner R, Walia R, Blackwell TS, Banovich NE, Kropski JA. Single-cell RNA sequencing reveals profibrotic roles of distinct epithelial and mesenchymal lineages in pulmonary fibrosis. Sci Adv 2020;6:eaba1972. 32832598 10.1126/sciadv.aba1972 PMC7439444

[55] Lambrechts D , Wauters E, Boeckx B, Aibar S, Nittner D, Burton O, Bassez A, Decaluwé H, Pircher A, Van den Eynde K, Weynand B, Verbeken E, De Leyn P, Liston A, Vansteenkiste J, Carmeliet P, Aerts S, Thienpont B. Phenotype molding of stromal cells in the lung tumor microenvironment. Nat Med 2018;24:1277–1289. 29988129 10.1038/s41591-018-0096-5

[56] Qian J , Olbrecht S, Boeckx B, Vos H, Laoui D, Etlioglu E, Wauters E, Pomella V, Verbandt S, Busschaert P, Bassez A, Franken A, Bempt MV, Xiong J, Weynand B, van Herck Y, Antoranz A, Bosisio FM, Thienpont B, Floris G, Vergote I, Smeets A, Tejpar S, Lambrechts D. A pan-cancer blueprint of the heterogeneous tumor microenvironment revealed by single-cell profiling. Cell Res 2020;30:745–762. 32561858 10.1038/s41422-020-0355-0 PMC7608385

[57] Reyfman PA , Walter JM, Joshi N, Anekalla KR, McQuattie-Pimentel AC, Chiu S, Fernandez R, Akbarpour M, Chen C-I, Ren Z, Verma R, Abdala-Valencia H, Nam K, Chi M, Han SHye, Gonzalez-Gonzalez FJ, Soberanes S, Watanabe S, Williams KJN, Flozak AS, Nicholson TT, Morgan VK, Winter DR, Hinchcliff M, Hrusch CL, Guzy RD, Bonham CA, Sperling AI, Bag R, Hamanaka RB, Mutlu GM, Yeldandi AV, Marshall SA, Shilatifard A, Amaral LAN, Perlman H, Sznajder JI, Argento AC, Gillespie CT, Dematte J, Jain M, Singer BD, Ridge KM, Lam AP, Bharat A, Bhorade SM, Gottardi CJ, Budinger GRS, Misharin AV. Single-cell transcriptomic analysis of human lung provides insights into the pathobiology of pulmonary fibrosis. Am J Respir Crit Care Med 2019;199:1517–1536. 30554520 10.1164/rccm.201712-2410OC PMC6580683

[58] Melms JC , Biermann J, Huang H, Wang Y, Nair A, Tagore S, Katsyv I, Rendeiro AF, Amin AD, Schapiro D, Frangieh CJ, Luoma AM, Filliol A, Fang Y, Ravichandran H, Clausi MG, Alba GA, Rogava M, Chen SW, Ho P, Montoro DT, Kornberg AE, Han AS, Bakhoum MF, Anandasabapathy N, Suárez-Fariñas M, Bakhoum SF, Bram Y, Borczuk A, Guo XV, Lefkowitch JH, Marboe C, Lagana SM, Del Portillo A, Tsai EJ, Zorn E, Markowitz GS, Schwabe RF, Schwartz RE, Elemento O, Saqi A, Hibshoosh H, Que J, Izar B. A molecular single-cell lung atlas of lethal COVID-19. Nature 2021;595:114–119. 33915568 10.1038/s41586-021-03569-1 PMC8814825

[59] Ramachandran P , Dobie R, Wilson-Kanamori JR, Dora EF, Henderson BEP, Luu NT, Portman JR, Matchett KP, Brice M, Marwick JA, Taylor RS, Efremova M, Vento-Tormo R, Carragher NO, Kendall TJ, Fallowfield JA, Harrison EM, Mole DJ, Wigmore SJ, Newsome PN, Weston CJ, Iredale JP, Tacke F, Pollard JW, Ponting CP, Marioni JC, Teichmann SA, Henderson NC. Resolving the fibrotic niche of human liver cirrhosis at single-cell level. Nature 2019;575:512–518. 31597160 10.1038/s41586-019-1631-3 PMC6876711

[60] Nicin L , Abplanalp WT, Mellentin H, Kattih B, Tombor L, John D, Schmitto JD, Heineke J, Emrich F, Arsalan M, Holubec T, Walther T, Zeiher AM, Dimmeler S. Cell type-specific expression of the putative SARS-CoV-2 receptor ACE2 in human hearts. Eur Heart J 2020;41:1804–1806. 32293672 10.1093/eurheartj/ehaa311 PMC7184464

[61] Wang L , Yu P, Zhou B, Song J, Li Z, Zhang M, Guo G, Wang Y, Chen X, Han L, Hu S. Single-cell reconstruction of the adult human heart during heart failure and recovery reveals the cellular landscape underlying cardiac function. Nat Cell Biol 2020;22:108–119. 31915373 10.1038/s41556-019-0446-7

[62] Suryawanshi H , Clancy R, Morozov P, Halushka MK, Buyon JP, Tuschl T. Cell atlas of the foetal human heart and implications for autoimmune-mediated congenital heart block. Cardiovasc Res 2020;116:1446–1457. 31589297 10.1093/cvr/cvz257 PMC7314636

[63] Nicin L , Abplanalp WT, Schänzer A, Sprengel A, John D, Mellentin H, Tombor L, Keuper M, Ullrich E, Klingel K, Dettmeyer RB, Hoffmann J, Akintuerk H, Jux C, Schranz D, Zeiher AM, Rupp S, Dimmeler S. Single nuclei sequencing reveals novel insights into the regulation of cellular signatures in children with dilated cardiomyopathy. Circulation 2021;143:1704–1719. 33618539 10.1161/CIRCULATIONAHA.120.051391

[64] Lau S-F , Cao H, Fu AKY, Ip NY. Single-nucleus transcriptome analysis reveals dysregulation of angiogenic endothelial cells and neuroprotective glia in Alzheimer’s disease. Proc Natl Acad Sci USA 2020;117:25800–25809. 32989152 10.1073/pnas.2008762117 PMC7568283

[65] Rohlenova K , Goveia J, García-Caballero M, Subramanian A, Kalucka J, Treps L, Falkenberg KD, de Rooij LPMH, Zheng Y, Lin L, Sokol L, Teuwen L-A, Geldhof V, Taverna F, Pircher A, Conradi L-C, Khan S, Stegen S, Panovska D, De Smet F, Staal FJT, Mclaughlin RJ, Vinckier S, Van Bergen T, Ectors N, De Haes P, Wang J, Bolund L, Schoonjans L, Karakach TK, Yang H, Carmeliet G, Liu Y, Thienpont B, Dewerchin M, Eelen G, Li X, Luo Y, Carmeliet P. Single-cell RNA sequencing maps endothelial metabolic plasticity in pathological angiogenesis. Cell Metab 2020;31:862–877.e14. 32268117 10.1016/j.cmet.2020.03.009

[66] Raredon MSB , Adams TS, Suhail Y, Schupp JC, Poli S, Neumark N, Leiby KL, Greaney AM, Yuan Y, Horien C, Linderman G, Engler AJ, Boffa DJ, Kluger Y, Rosas IO, Levchenko A, Kaminski N, Niklason LE. Single-cell connectomic analysis of adult mammalian lungs. Sci Adv 2019;5:eaaw3851. 31840053 10.1126/sciadv.aaw3851 PMC6892628

[67] Baryawno N , Przybylski D, Kowalczyk MS, Kfoury Y, Severe N, Gustafsson K, Kokkaliaris KD, Mercier F, Tabaka M, Hofree M, Dionne D, Papazian A, Lee D, Ashenberg O, Subramanian A, Vaishnav ED, Rozenblatt-Rosen O, Regev A, Scadden DT. A cellular taxonomy of the bone marrow stroma in homeostasis and leukemia. Cell 2019;177:1915–1932.e16. 31130381 10.1016/j.cell.2019.04.040 PMC6570562

[68] Gillich A , Zhang F, Farmer CG, Travaglini KJ, Tan SY, Gu M, Zhou B, Feinstein JA, Krasnow MA, Metzger RJ. Capillary cell-type specialization in the alveolus. Nature 2020;586:785–789. 33057196 10.1038/s41586-020-2822-7 PMC7721049

[69] Travaglini KJ , Nabhan AN, Penland L, Sinha R, Gillich A, Sit RV, Chang S, Conley SD, Mori Y, Seita J, Berry GJ, Shrager JB, Metzger RJ, Kuo CS, Neff N, Weissman IL, Quake SR, Krasnow MA. A molecular cell atlas of the human lung from single-cell RNA sequencing. Nature 2020;587:619–625. 33208946 10.1038/s41586-020-2922-4 PMC7704697

[70] Vanlandewijck M , Betsholtz C. Single-cell mRNA sequencing of the mouse brain vasculature. Methods Mol Biol 2018;1846:309–324. 30242769 10.1007/978-1-4939-8712-2_21

[71] Banks WA. From blood-brain barrier to blood-brain interface: new opportunities for CNS drug delivery. Nat Rev Drug Discov 2016;15:275–292. 26794270 10.1038/nrd.2015.21

[72] Brandes RP , Fleming I, Busse R. Endothelial aging. Cardiovasc Res 2005;66:286–294. 15820197 10.1016/j.cardiores.2004.12.027

[73] Vidal R , Wagner JUG, Braeuning C, Fischer C, Patrick R, Tombor L, Muhly-Reinholz M, John D, Kliem M, Conrad T, Guimarães-Camboa N, Harvey R, Dimmeler S, Sauer S. Transcriptional heterogeneity of fibroblasts is a hallmark of the aging heart. JCI Insight 2019;4:e131092. 31723062 10.1172/jci.insight.131092 PMC6948853

[74] Zhao L , Li Z, Vong JSL, Chen X, Lai H-M, Yan LYC, Huang J, Sy SKH, Tian X, Huang Y, Chan HYE, So H-C, Ng W-L, Tang Y, Lin W-J, Mok VCT, Ko H. Pharmacologically reversible zonation-dependent endothelial cell transcriptomic changes with neurodegenerative disease associations in the aged brain. Nat Commun 2020;11:4413. 32887883 10.1038/s41467-020-18249-3 PMC7474063

[75] de Soysa TY , Ranade SS, Okawa S, Ravichandran S, Huang Y, Salunga HT, Schricker A, Del Sol A, Gifford CA, Srivastava D. Single-cell analysis of cardiogenesis reveals basis for organ-level developmental defects. Nature 2019;572:120–124. 31341279 10.1038/s41586-019-1414-x PMC6719697

[76] Zhang H , Lui KO, Zhou B. Endocardial cell plasticity in cardiac development, diseases and regeneration. Circ Res 2018;122:774–789. 29496799 10.1161/CIRCRESAHA.117.312136

[77] de Lange FJ , Moorman AFM, Anderson RH, Männer J, Soufan AT, de Gier-de Vries C, Schneider MD, Webb S, van den Hoff MJB, Christoffels VM. Lineage and morphogenetic analysis of the cardiac valves. Circ Res 2004;95:645–654. 15297379 10.1161/01.RES.0000141429.13560.cb

[78] Ibarra-Soria X , Jawaid W, Pijuan-Sala B, Ladopoulos V, Scialdone A, Jörg DJ, Tyser RCV, Calero-Nieto FJ, Mulas C, Nichols J, Vallier L, Srinivas S, Simons BD, Göttgens B, Marioni JC. Defining murine organogenesis at single-cell resolution reveals a role for the leukotriene pathway in regulating blood progenitor formation. Nat Cell Biol 2018;20:127–134. 29311656 10.1038/s41556-017-0013-z PMC5787369

[79] Briggs JA , Weinreb C, Wagner DE, Megason S, Peshkin L, Kirschner MW, Klein AM. The dynamics of gene expression in vertebrate embryogenesis at single-cell resolution. Science 2018;360:eaar5780. 29700227 10.1126/science.aar5780 PMC6038144

[80] Vila Ellis L , Cain MP, Hutchison V, Flodby P, Crandall ED, Borok Z, Zhou B, Ostrin EJ, Wythe JD, Chen J. Epithelial Vegfa specifies a distinct endothelial population in the mouse lung. Dev Cell 2020;52:617–630.e16. 32059772 10.1016/j.devcel.2020.01.009 PMC7170573

[81] Boisset J-C , van Cappellen W, Andrieu-Soler C, Galjart N, Dzierzak E, Robin C. *In vivo* imaging of haematopoietic cells emerging from the mouse aortic endothelium. Nature 2010;464:116–120. 20154729 10.1038/nature08764

[82] Zovein AC , Hofmann JJ, Lynch M, French WJ, Turlo KA, Yang Y, Becker MS, Zanetta L, Dejana E, Gasson JC, Tallquist MD, Iruela-Arispe ML. Fate tracing reveals the endothelial origin of hematopoietic stem cells. Cell Stem Cell 2008;3:625–636. 19041779 10.1016/j.stem.2008.09.018 PMC2631552

[83] Ema M , Yokomizo T, Wakamatsu A, Terunuma T, Yamamoto M, Takahashi S. Primitive erythropoiesis from mesodermal precursors expressing VE-cadherin, PECAM-1, Tie2, endoglin, and CD34 in the mouse embryo. Blood 2006;108:4018–4024. 16926294 10.1182/blood-2006-03-012872

[84] Pijuan-Sala B , Wilson NK, Xia J, Hou X, Hannah RL, Kinston S, Calero-Nieto FJ, Poirion O, Preissl S, Liu F, Göttgens B. Single-cell chromatin accessibility maps reveal regulatory programs driving early mouse organogenesis. Nat Cell Biol 2020;22:487–497. 32231307 10.1038/s41556-020-0489-9 PMC7145456

[85] Rao M , Oh K, Moffitt R, Thompson P, Li J, Liu J, Sasson A, Georgakis G, Kim J, Choi M, Powers S. Comparative single-cell RNA sequencing (scRNA-seq) reveals liver metastasis-specific targets in a patient with small intestinal neuroendocrine cancer. Cold Spring Harb Mol Case Stud 2020;6:a004978. 32054662 10.1101/mcs.a004978 PMC7133744

[86] Reynolds G , Vegh P, Fletcher J, Poyner EFM, Stephenson E, Goh I, Botting RA, Huang N, Olabi B, Dubois A, Dixon D, Green K, Maunder D, Engelbert J, Efremova M, Polański K, Jardine L, Jones C, Ness T, Horsfall D, McGrath J, Carey C, Popescu D-M, Webb S, Wang X-N, Sayer B, Park J-E, Negri VA, Belokhvostova D, Lynch MD, McDonald D, Filby A, Hagai T, Meyer KB, Husain A, Coxhead J, Vento-Tormo R, Behjati S, Lisgo S, Villani A-C, Bacardit J, Jones PH, O’Toole EA, Ogg GS, Rajan N, Reynolds NJ, Teichmann SA, Watt FM, Haniffa M. Developmental cell programs are co-opted in inflammatory skin disease. Science 2021;371:eaba6500. 33479125 10.1126/science.aba6500 PMC7611557

[87] Hurskainen M , Mižíková I, Cook DP, Andersson N, Cyr-Depauw C, Lesage F, Helle E, Renesme L, Jankov RP, Heikinheimo M, Vanderhyden BC, Thébaud B. Single cell transcriptomic analysis of murine lung development on hyperoxia-induced damage. Nat Commun 2021;12:1565. 33692365 10.1038/s41467-021-21865-2 PMC7946947

[88] Muhleder S , Fernandez-Chacon M, Garcia-Gonzalez I, Benedito R. Endothelial sprouting, proliferation, or senescence: tipping the balance from physiology to pathology. Cell Mol Life Sci 2021;78:1329–1354. 33078209 10.1007/s00018-020-03664-y PMC7904752

[89] Li Z , Solomonidis EG, Meloni M, Taylor RS, Duffin R, Dobie R, Magalhaes MS, Henderson BEP, Louwe PA, D'Amico G, Hodivala-Dilke KM, Shah AM, Mills NL, Simons BD, Gray GA, Henderson NC, Baker AH, Brittan M. Single-cell transcriptome analyses reveal novel targets modulating cardiac neovascularization by resident endothelial cells following myocardial infarction. Eur Heart J 2019;40:2507–2520. 31162546 10.1093/eurheartj/ehz305 PMC6685329

[90] Niethamer TK , Stabler CT, Leach JP, Zepp JA, Morley MP, Babu A, Zhou S, Morrisey EE. Defining the role of pulmonary endothelial cell heterogeneity in the response to acute lung injury. Elife 2020;9:e53072. 32091393 10.7554/eLife.53072 PMC7176435

[91] Manavski Y , Lucas T, Glaser SF, Dorsheimer L, Günther S, Braun T, Rieger MA, Zeiher AM, Boon RA, Dimmeler S. Clonal expansion of endothelial cells contributes to ischemia-induced neovascularization. Circ Res 2018;122:670–677. 29358229 10.1161/CIRCRESAHA.117.312310

[92] Wakabayashi T , Naito H, Suehiro J-I, Lin Y, Kawaji H, Iba T, Kouno T, Ishikawa-Kato S, Furuno M, Takara K, Muramatsu F, Weizhen J, Kidoya H, Ishihara K, Hayashizaki Y, Nishida K, Yoder MC, Takakura N. CD157 marks tissue-resident endothelial stem cells with homeostatic and regenerative properties. Cell Stem Cell 2018;22:384–397.e6. 29429943 10.1016/j.stem.2018.01.010

[93] McDonald AI , Shirali AS, Aragón R, Ma F, Hernandez G, Vaughn DA, Mack JJ, Lim TY, Sunshine H, Zhao P, Kalinichenko V, Hai T, Pelegrini M, Ardehali R, Iruela-Arispe ML. Endothelial regeneration of large vessels is a biphasic process driven by local cells with distinct proliferative capacities. Cell Stem Cell 2018;23:210–225.e16. 30075129 10.1016/j.stem.2018.07.011 PMC6178982

[94] Guo L , Zhang H, Hou Y, Wei T, Liu J. Plasmalemma vesicle-associated protein: a crucial component of vascular homeostasis. Exp Ther Med 2016;12:1639–1644. 27602081 10.3892/etm.2016.3557 PMC4998186

[95] Chen S , Zhu G, Yang Y, Wang F, Xiao Y-T, Zhang N, Bian X, Zhu Y, Yu Y, Liu F, Dong K, Mariscal J, Liu Y, Soares F, Loo Yau H, Zhang B, Chen W, Wang C, Chen D, Guo Q, Yi Z, Liu M, Fraser M, De Carvalho DD, Boutros PC, Di Vizio D, Jiang Z, van der Kwast T, Berlin A, Wu S, Wang J, He HH, Ren S. Single-cell analysis reveals transcriptomic remodellings in distinct cell types that contribute to human prostate cancer progression. Nat Cell Biol 2021;23:87–98. 33420488 10.1038/s41556-020-00613-6

[96] Carmeliet P , De Smet F, Loges S, Mazzone M. Branching morphogenesis and antiangiogenesis candidates: tip cells lead the way. Nat Rev Clin Oncol 2009;6:315–326. 19483738 10.1038/nrclinonc.2009.64

[97] Orsenigo F , Conze LL, Jauhiainen S, Corada M, Lazzaroni F, Malinverno M, Sundell V, Cunha SI, Brännström J, Globisch MA, Maderna C, Lampugnani MG, Magnusson PU, Dejana E. Mapping endothelial-cell diversity in cerebral cavernous malformations at single-cell resolution. Elife 2020;9:e61413. 33138917 10.7554/eLife.61413 PMC7609066

[98] Kovacic JC , Dimmeler S, Harvey RP, Finkel T, Aikawa E, Krenning G, Baker AH. Endothelial to mesenchymal transition in cardiovascular disease: JACC state-of-the-art review. J Am Coll Cardiol 2019;73:190–209. 30654892 10.1016/j.jacc.2018.09.089 PMC6865825

[99] Hong L , Du X, Li W, Mao Y, Sun L, Li X. EndMT: a promising and controversial field. Eur J Cell Biol 2018;97:493–500. 30082099 10.1016/j.ejcb.2018.07.005

[100] Su T , Yang Y, Lai S, Jeong J, Jung Y, McConnell M, Utsumi T, Iwakiri Y. Single-cell transcriptomics reveals zone-specific alterations of liver sinusoidal endothelial cells in cirrhosis. Cell Mol Gastroenterol Hepatol 2021;11:1139–1161. 33340713 10.1016/j.jcmgh.2020.12.007 PMC7903131

[101] Xu K , Xie S, Huang Y, Zhou T, Liu M, Zhu P, Wang C, Shi J, Li F, Sellke FW, Dong N. Cell-type transcriptome atlas of human aortic valves reveal cell heterogeneity and endothelial to mesenchymal transition involved in calcific aortic valve disease. Arterioscler Thromb Vasc Biol 2020;40:2910–2921. 33086873 10.1161/ATVBAHA.120.314789

[102] Chen P-Y , Qin L, Li G, Wang Z, Dahlman JE, Malagon-Lopez J, Gujja S, Cilfone NA, Kauffman KJ, Sun L, Sun H, Zhang X, Aryal B, Canfran-Duque A, Liu R, Kusters P, Sehgal A, Jiao Y, Anderson DG, Gulcher J, Fernandez-Hernando C, Lutgens E, Schwartz MA, Pober JS, Chittenden TW, Tellides G, Simons M. Endothelial TGF-beta signalling drives vascular inflammation and atherosclerosis. Nat Metab 2019;1:912–926. 31572976 10.1038/s42255-019-0102-3 PMC6767930

[103] Tombor LS , John D, Glaser SF, Luxán G, Forte E, Furtado M, Rosenthal N, Baumgarten N, Schulz MH, Wittig J, Rogg E-M, Manavski Y, Fischer A, Muhly-Reinholz M, Klee K, Looso M, Selignow C, Acker T, Bibli S-I, Fleming I, Patrick R, Harvey RP, Abplanalp WT, Dimmeler S. Single cell sequencing reveals endothelial plasticity with transient mesenchymal activation after myocardial infarction. Nat Commun 2021;12:681. 33514719 10.1038/s41467-021-20905-1 PMC7846794

[104] Veerman K , Tardiveau C, Martins F, Coudert J, Girard JP. Single-cell analysis reveals heterogeneity of high endothelial venules and different regulation of genes controlling lymphocyte entry to lymph nodes. Cell Rep 2019;26:3116–3131.e15. 30865898 10.1016/j.celrep.2019.02.042

[105] Rodor J , Chen SH, Scanlon JP, Monteiro JP, Caudrillier A, Sweta S, Stewart KR, Shmakova A, Dobie R, Henderson BEP, Stewart K, Hadoke PWF, Southwood M, Moore SD, Upton PD, Morrell NW, Li Z, Chan SY, Handen A, Lafyatis R, de Rooij LPMH, Henderson NC, Carmeliet P, Spiroski AM, Brittan M, Baker AH. Single-cell RNA sequencing profiling of mouse endothelial cells in response to pulmonary arterial hypertension. Cardiovasc Res 2022;118:2519–2534. 34528097 10.1093/cvr/cvab296 PMC9400412

[106] Pasut A , Becker LM, Cuypers A, Carmeliet P. Endothelial cell plasticity at the single-cell level. Angiogenesis 2021;24:311–326. 34061284 10.1007/s10456-021-09797-3 PMC8169404

[107] Apostolidis SA , Stifano G, Tabib T, Rice LM, Morse CM, Kahaleh B, Lafyatis R. Single cell RNA sequencing identifies HSPG2 and APLNR as markers of endothelial cell injury in systemic sclerosis skin. Front Immunol 2018;9:2191. 30327649 10.3389/fimmu.2018.02191 PMC6174292

[108] Crespo-Garcia S , Tsuruda PR, Dejda A, Ryan RD, Fournier F, Chaney SY, Pilon F, Dogan T, Cagnone G, Patel P, Buscarlet M, Dasgupta S, Girouard G, Rao SR, Wilson AM, O'Brien R, Juneau R, Guber V, Dubrac A, Beausejour C, Armstrong S, Mallette FA, Yohn CB, Joyal J-S, Marquess D, Beltran PJ, Sapieha P. Pathological angiogenesis in retinopathy engages cellular senescence and is amenable to therapeutic elimination via BCL-xL inhibition. Cell Metab 2021;33:818–832.e17. 33548171 10.1016/j.cmet.2021.01.011

[109] Aibar S , González-Blas CB, Moerman T, Huynh-Thu VA, Imrichova H, Hulselmans G, Rambow F, Marine J-C, Geurts P, Aerts J, van den Oord J, Atak ZK, Wouters J, Aerts S. SCENIC: single-cell regulatory network inference and clustering. Nat Methods 2017;14:1083–1086. 28991892 10.1038/nmeth.4463 PMC5937676

[110] Holland CH , Tanevski J, Perales-Patón J, Gleixner J, Kumar MP, Mereu E, Joughin BA, Stegle O, Lauffenburger DA, Heyn H, Szalai B, Saez-Rodriguez J. Robustness and applicability of transcription factor and pathway analysis tools on single-cell RNA-seq data. Genome Biol 2020;21:36. 32051003 10.1186/s13059-020-1949-z PMC7017576

[111] Minami T , Muramatsu M, Kume T. Organ/tissue-specific vascular endothelial cell heterogeneity in health and disease. Biol Pharm Bull 2019;42:1609–1619. 31582649 10.1248/bpb.b19-00531

[112] Yang Y , Guo Z, Chen W, Wang X, Cao M, Han X, Zhang K, Teng B, Cao J, Wu W, Cao P, Huang C, Qiu Z. M2 macrophage-derived exosomes promote angiogenesis and growth of pancreatic ductal adenocarcinoma by targeting E2F2. Mol Ther 2021;29:1226–1238. 33221435 10.1016/j.ymthe.2020.11.024 PMC7934635

[113] Choi H , Moon A. Crosstalk between cancer cells and endothelial cells: implications for tumor progression and intervention. Arch Pharm Res 2018;41:711–724. 29961196 10.1007/s12272-018-1051-1

[114] Colliva A , Braga L, Giacca M, Zacchigna S. Endothelial cell-cardiomyocyte crosstalk in heart development and disease. J Physiol 2020;598:2923–2939. 30816576 10.1113/JP276758 PMC7496632

[115] Wan A , Rodrigues B. Endothelial cell–cardiomyocyte crosstalk in diabetic cardiomyopathy. Cardiovasc Res 2016;111:172–183. 27288009 10.1093/cvr/cvw159 PMC4957492

[116] Efremova M , Vento-Tormo M, Teichmann SA, Vento-Tormo R. CellPhoneDB: inferring cell-cell communication from combined expression of multi-subunit ligand-receptor complexes. Nat Protoc 2020;15:1484–1506. 32103204 10.1038/s41596-020-0292-x

[117] Jin S , Guerrero-Juarez CF, Zhang L, Chang I, Ramos R, Kuan C-H, Myung P, Plikus MV, Nie Q. Inference and analysis of cell-cell communication using CellChat. Nat Commun 2021;12:1088. 33597522 10.1038/s41467-021-21246-9 PMC7889871

[118] Browaeys R , Saelens W, Saeys Y. NicheNet: modeling intercellular communication by linking ligands to target genes. Nat Methods 2020;17:159–162. 31819264 10.1038/s41592-019-0667-5

[119] Farbehi N , Patrick R, Dorison A, Xaymardan M, Janbandhu V, Wystub-Lis K, Ho JW, Nordon RE, Harvey RP. Single-cell expression profiling reveals dynamic flux of cardiac stromal, vascular and immune cells in health and injury. Elife 2019;8:e43882. 30912746 10.7554/eLife.43882 PMC6459677

[120] Cook KM , Figg WD. Angiogenesis inhibitors: current strategies and future prospects. CA Cancer J Clin 2010;60:222–243. 20554717 10.3322/caac.20075 PMC2919227

[121] Potente M , Gerhardt H, Carmeliet P. Basic and therapeutic aspects of angiogenesis. Cell 2011;146:873–887. 21925313 10.1016/j.cell.2011.08.039

[122] Folkman J. Tumor angiogenesis: therapeutic implications. N Engl J Med 1971;285:1182–1186. 4938153 10.1056/NEJM197111182852108

[123] Yuan F , Chen Y, Dellian M, Safabakhsh N, Ferrara N, Jain RK. Time-dependent vascular regression and permeability changes in established human tumor xenografts induced by an anti-vascular endothelial growth factor/vascular permeability factor antibody. Proc Natl Acad Sci USA 1996;93:14765–14770. 8962129 10.1073/pnas.93.25.14765 PMC26210

[124] Kim KJ , Li B, Winer J, Armanini M, Gillett N, Phillips HS, Ferrara N. Inhibition of vascular endothelial growth factor-induced angiogenesis suppresses tumour growth *in vivo*. Nature 1993;362:841–844. 7683111 10.1038/362841a0

[125] Eelen G , Treps L, Li X, Carmeliet P. Basic and therapeutic aspects of angiogenesis updated. Circ Res 2020;127:310–329. 32833569 10.1161/CIRCRESAHA.120.316851

[126] De Bock K , Mazzone M, Carmeliet P. Antiangiogenic therapy, hypoxia, and metastasis: risky liaisons, or not? Nat Rev Clin Oncol 2011;8:393–404. 21629216 10.1038/nrclinonc.2011.83

[127] Loges S , Mazzone M, Hohensinner P, Carmeliet P. Silencing or fueling metastasis with VEGF inhibitors: antiangiogenesis revisited. Cancer Cell 2009;15:167–170. 19249675 10.1016/j.ccr.2009.02.007

[128] Bottsford-Miller JN , Coleman RL, Sood AK. Resistance and escape from antiangiogenesis therapy: clinical implications and future strategies. J Clin Oncol 2012;30:4026–4034. 23008289 10.1200/JCO.2012.41.9242 PMC3488272

[129] Sennino B , McDonald DM. Controlling escape from angiogenesis inhibitors. Nat Rev Cancer 2012;12:699–709. 23001349 10.1038/nrc3366 PMC3969886

[130] Teuwen L-A , De Rooij LPMH, Cuypers A, Rohlenova K, Dumas SJ, García-Caballero M, Meta E, Amersfoort J, Taverna F, Becker LM, Veiga N, Cantelmo AR, Geldhof V, Conchinha NV, Kalucka J, Treps L, Conradi L-C, Khan S, Karakach TK, Soenen S, Vinckier S, Schoonjans L, Eelen G, Van Laere S, Dewerchin M, Dirix L, Mazzone M, Luo Y, Vermeulen P, Carmeliet P. Tumor vessel co-option probed by single-cell analysis. Cell Rep 2021;35:109253. 34133923 10.1016/j.celrep.2021.109253

[131] Bridgeman VL , Vermeulen PB, Foo S, Bilecz A, Daley F, Kostaras E, Nathan MR, Wan E, Frentzas S, Schweiger T, Hegedus B, Hoetzenecker K, Renyi-Vamos F, Kuczynski EA, Vasudev NS, Larkin J, Gore M, Dvorak HF, Paku S, Kerbel RS, Dome B, Reynolds AR. Vessel co-option is common in human lung metastases and mediates resistance to anti-angiogenic therapy in preclinical lung metastasis models. J Pathol 2017;241:362–374. 27859259 10.1002/path.4845 PMC5248628

[132] Zhao Q , Eichten A, Parveen A, Adler C, Huang Y, Wang W, Ding Y, Adler A, Nevins T, Ni M, Wei Y, Thurston G. Single-cell transcriptome analyses reveal endothelial cell heterogeneity in tumors and changes following antiangiogenic treatment. Cancer Res 2018;78:2370–2382. 29449267 10.1158/0008-5472.CAN-17-2728

[133] Kim N , Kim HK, Lee K, Hong Y, Cho JH, Choi JW, Lee J-I, Suh Y-L, Ku BM, Eum HH, Choi S, Choi Y-L, Joung J-G, Park W-Y, Jung HA, Sun J-M, Lee S-H, Ahn JS, Park K, Ahn M-J, Lee H-O. Single-cell RNA sequencing demonstrates the molecular and cellular reprogramming of metastatic lung adenocarcinoma. Nat Commun 2020;11:2285. 32385277 10.1038/s41467-020-16164-1 PMC7210975

[134] Hida K , Maishi N, Akiyama K, Ohmura-Kakutani H, Torii C, Ohga N, Osawa T, Kikuchi H, Morimoto H, Morimoto M, Shindoh M, Shinohara N, Hida Y. Tumor endothelial cells with high aldehyde dehydrogenase activity show drug resistance. Cancer Sci 2017;108:2195–2203. 28851003 10.1111/cas.13388 PMC5666026

[135] Ohmura-Kakutani H , Akiyama K, Maishi N, Ohga N, Hida Y, Kawamoto T, Iida J, Shindoh M, Tsuchiya K, Shinohara N, Hida K. Identification of tumor endothelial cells with high aldehyde dehydrogenase activity and a highly angiogenic phenotype. PLoS One 2014;9:e113910. 25437864 10.1371/journal.pone.0113910 PMC4250080

[136] Hlushchuk R , Riesterer O, Baum O, Wood J, Gruber G, Pruschy M, Djonov V. Tumor recovery by angiogenic switch from sprouting to intussusceptive angiogenesis after treatment with PTK787/ZK222584 or ionizing radiation. Am J Pathol 2008;173:1173–1185. 18787105 10.2353/ajpath.2008.071131 PMC2543084

[137] Angara K , Rashid MH, Shankar A, Ara R, Iskander A, Borin TF, Jain M, Achyut BR, Arbab AS. Vascular mimicry in glioblastoma following anti-angiogenic and anti-20-HETE therapies. Histol Histopathol 2017;32:917–928. 27990624 10.14670/HH-11-856 PMC5476524

[138] James MD , Andrew CD. Vascular mimicry: concepts and implications for anti-angiogenic therapy. Curr Angiogenes 2012;1:133–138. 24729954 10.2174/2211552811201020133 PMC3982611

[139] Zirlik K , Duyster J. Anti-angiogenics: current situation and future perspectives. Oncol Res Treat 2018;41:166–171. 29562226 10.1159/000488087

[140] Rini BI , Bellmunt J, Clancy J, Wang K, Niethammer AG, Hariharan S, Escudier B. Randomized phase III trial of temsirolimus and bevacizumab versus interferon alfa and bevacizumab in metastatic renal cell carcinoma: INTORACT trial. J Clin Oncol 2014;32:752–759. 24297945 10.1200/JCO.2013.50.5305

[141] Ager A , May MJ. Understanding high endothelial venules: lessons for cancer immunology. Oncoimmunology 2015;4:e1008791. 26155419 10.1080/2162402X.2015.1008791 PMC4485764

[142] De Bock K , Georgiadou M, Schoors S, Kuchnio A, Wong BW, Cantelmo AR, Quaegebeur A, Ghesquière B, Cauwenberghs S, Eelen G, Phng L-K, Betz I, Tembuyser B, Brepoels K, Welti J, Geudens I, Segura I, Cruys B, Bifari F, Decimo I, Blanco R, Wyns S, Vangindertael J, Rocha S, Collins RT, Munck S, Daelemans D, Imamura H, Devlieger R, Rider M, Van Veldhoven PP, Schuit F, Bartrons R, Hofkens J, Fraisl P, Telang S, Deberardinis RJ, Schoonjans L, Vinckier S, Chesney J, Gerhardt H, Dewerchin M, Carmeliet P. Role of PFKFB3-driven glycolysis in vessel sprouting. Cell 2013;154:651–663. 23911327 10.1016/j.cell.2013.06.037

[143] Dumas SJ , García-Caballero M, Carmeliet P. Metabolic signatures of distinct endothelial phenotypes. Trends Endocrinol Metab 2020;31:580–595. 32622584 10.1016/j.tem.2020.05.009

[144] Schoors S , De Bock K, Cantelmo AR, Georgiadou M, Ghesquière B, Cauwenberghs S, Kuchnio A, Wong BW, Quaegebeur A, Goveia J, Bifari F, Wang X, Blanco R, Tembuyser B, Cornelissen I, Bouché A, Vinckier S, Diaz-Moralli S, Gerhardt H, Telang S, Cascante M, Chesney J, Dewerchin M, Carmeliet P. Partial and transient reduction of glycolysis by PFKFB3 blockade reduces pathological angiogenesis. Cell Metab 2014;19:37–48. 24332967 10.1016/j.cmet.2013.11.008

[145] Schoors S , Bruning U, Missiaen R, Queiroz KC, Borgers G, Elia I, Zecchin A, Cantelmo AR, Christen S, Goveia J, Heggermont W, Goddé L, Vinckier S, Van Veldhoven PP, Eelen G, Schoonjans L, Gerhardt H, Dewerchin M, Baes M, De Bock K, Ghesquière B, Lunt SY, Fendt S-M, Carmeliet P. Fatty acid carbon is essential for dNTP synthesis in endothelial cells. Nature 2015;520:192–197. 25830893 10.1038/nature14362 PMC4413024

[146] Kalucka J , Bierhansl L, Conchinha NV, Missiaen R, Elia I, Brüning U, Scheinok S, Treps L, Cantelmo AR, Dubois C, de Zeeuw P, Goveia J, Zecchin A, Taverna F, Morales-Rodriguez F, Brajic A, Conradi L-C, Schoors S, Harjes U, Vriens K, Pilz G-A, Chen R, Cubbon R, Thienpont B, Cruys B, Wong BW, Ghesquière B, Dewerchin M, De Bock K, Sagaert X, Jessberger S, Jones EAV, Gallez B, Lambrechts D, Mazzone M, Eelen G, Li X, Fendt S-M, Carmeliet P. Quiescent endothelial cells upregulate fatty acid beta-oxidation for vasculoprotection via redox homeostasis. Cell Metab 2018;28:881–894.e13. 30146488 10.1016/j.cmet.2018.07.016

[147] Eelen G , de Zeeuw P, Treps L, Harjes U, Wong BW, Carmeliet P. Endothelial cell metabolism. Physiol Rev 2018;98:3–58. 29167330 10.1152/physrev.00001.2017 PMC5866357

[148] Teuwen LA , Geldhof V, Carmeliet P. How glucose, glutamine and fatty acid metabolism shape blood and lymph vessel development. Dev Biol 2019;447:90–102. 29224892 10.1016/j.ydbio.2017.12.001

[149] Lu T , Yang X, Shi Y, Zhao M, Bi G, Liang J, Chen Z, Huang Y, Jiang W, Lin Z, Xi J, Wang S, Yang Y, Zhan C, Wang Q, Tan L. Single-cell transcriptome atlas of lung adenocarcinoma featured with ground glass nodules. Cell Discov 2020;6:69. 33083004 10.1038/s41421-020-00200-x PMC7536439

[150] Rivello F , Matuła K, Piruska A, Smits M, Mehra N, Huck WTS. Probing single-cell metabolism reveals prognostic value of highly metabolically active circulating stromal cells in prostate cancer. Sci Adv 2020;6:eaaz3849. 32998889 10.1126/sciadv.aaz3849 PMC7527228

[151] Cantelmo AR , Conradi L-C, Brajic A, Goveia J, Kalucka J, Pircher A, Chaturvedi P, Hol J, Thienpont B, Teuwen L-A, Schoors S, Boeckx B, Vriens J, Kuchnio A, Veys K, Cruys B, Finotto L, Treps L, Stav-Noraas TE, Bifari F, Stapor P, Decimo I, Kampen K, De Bock K, Haraldsen G, Schoonjans L, Rabelink T, Eelen G, Ghesquière B, Rehman J, Lambrechts D, Malik AB, Dewerchin M, Carmeliet P. Inhibition of the glycolytic activator PFKFB3 in endothelium induces tumor vessel normalization, impairs metastasis, and improves chemotherapy. Cancer Cell 2016;30:968–985. 27866851 10.1016/j.ccell.2016.10.006 PMC5675554

[152] Bruning U , Morales-Rodriguez F, Kalucka J, Goveia J, Taverna F, Queiroz KCS, Dubois C, Cantelmo AR, Chen R, Loroch S, Timmerman E, Caixeta V, Bloch K, Conradi L-C, Treps L, Staes A, Gevaert K, Tee A, Dewerchin M, Semenkovich CF, Impens F, Schilling B, Verdin E, Swinnen JV, Meier JL, Kulkarni RA, Sickmann A, Ghesquière B, Schoonjans L, Li X, Mazzone M, Carmeliet P. Impairment of angiogenesis by fatty acid synthase inhibition involves mTOR malonylation. Cell Metab 2018;28:866–880.e15. 30146486 10.1016/j.cmet.2018.07.019 PMC8057116

[153] Seguin F , Carvalho MA, Bastos DC, Agostini M, Zecchin KG, Alvarez-Flores MP, Chudzinski-Tavassi AM, Coletta RD, Graner E. The fatty acid synthase inhibitor orlistat reduces experimental metastases and angiogenesis in B16-F10 melanomas. Br J Cancer 2012;107:977–987. 22892389 10.1038/bjc.2012.355 PMC3464771

[154] Calderon D , Bhaskar A, Knowles DA, Golan D, Raj T, Fu AQ, Pritchard JK. Inferring relevant cell types for complex traits by using single-cell gene expression. Am J Hum Genet 2017;101:686–699. 29106824 10.1016/j.ajhg.2017.09.009 PMC5673624

[155] Sierksma A , Lu A, Mancuso R, Fattorelli N, Thrupp N, Salta E, Zoco J, Blum D, Buée L, De Strooper B, Fiers M. Novel Alzheimer risk genes determine the microglia response to amyloid-β but not to TAU pathology. EMBO Mol Med 2020;12:e10606. 31951107 10.15252/emmm.201910606 PMC7059012

[156] Hong J , Arneson D, Umar S, Ruffenach G, Cunningham CM, Ahn IS, Diamante G, Bhetraratana M, Park JF, Said E, Huynh C, Le T, Medzikovic L, Humbert M, Soubrier F, Montani D, Girerd B, Trégouët D-A, Channick R, Saggar R, Eghbali M, Yang X. Single-cell study of two rat models of pulmonary arterial hypertension reveals connections to human pathobiology and drug repositioning. Am J Respir Crit Care Med 2021;203:1006–1022. 33021809 10.1164/rccm.202006-2169OC PMC8048757

[157] Lamb J , Crawford ED, Peck D, Modell JW, Blat IC, Wrobel MJ, Lerner J, Brunet J-P, Subramanian A, Ross KN, Reich M, Hieronymus H, Wei G, Armstrong SA, Haggarty SJ, Clemons PA, Wei R, Carr SA, Lander ES, Golub TR. The Connectivity Map: using gene-expression signatures to connect small molecules, genes, and disease. Science 2006;313:1929–1935. 17008526 10.1126/science.1132939

[158] Skinnider MA , Squair JW, Kathe C, Anderson MA, Gautier M, Matson KJE, Milano M, Hutson TH, Barraud Q, Phillips AA, Foster LJ, La Manno G, Levine AJ, Courtine G. Cell type prioritization in single-cell data. Nat Biotechnol 2021;39:30–34. 32690972 10.1038/s41587-020-0605-1 PMC7610525

[159] Gawel DR , Serra-Musach J, Lilja S, Aagesen J, Arenas A, Asking B, Bengnér M, Björkander J, Biggs S, Ernerudh J, Hjortswang H, Karlsson J-E, Köpsen M, Lee EJ, Lentini A, Li X, Magnusson M, Martínez-Enguita D, Matussek A, Nestor CE, Schäfer S, Seifert O, Sonmez C, Stjernman H, Tjärnberg A, Wu S, Åkesson K, Shalek AK, Stenmarker M, Zhang H, Gustafsson M, Benson M. A validated single-cell-based strategy to identify diagnostic and therapeutic targets in complex diseases. Genome Med 2019;11:47. 31358043 10.1186/s13073-019-0657-3 PMC6664760

[160] Jakab M , Augustin HG. Understanding angiodiversity: insights from single cell biology. Development 2020;147:dev146621. 32792338 10.1242/dev.146621

[161] Gauberti M , Montagne A, Marcos-Contreras OA, Le Béhot A, Maubert E, Vivien D. Ultra-sensitive molecular MRI of vascular cell adhesion molecule-1 reveals a dynamic inflammatory penumbra after strokes. Stroke 2013;44:1988–1996. 23743972 10.1161/STROKEAHA.111.000544

[162] Patel N , Duffy BA, Badar A, Lythgoe MF, Årstad E. Bimodal imaging of inflammation with SPECT/CT and MRI using iodine-125 labeled VCAM-1 targeting microparticle conjugates. Bioconjug Chem 2015;26:1542–1549. 26218622 10.1021/acs.bioconjchem.5b00380

[163] Marcos-Contreras OA , Greineder CF, Kiseleva RY, Parhiz H, Walsh LR, Zuluaga-Ramirez V, Myerson JW, Hood ED, Villa CH, Tombacz I, Pardi N, Seliga A, Mui BL, Tam YK, Glassman PM, Shuvaev VV, Nong J, Brenner JS, Khoshnejad M, Madden T, Weissmann D, Persidsky Y, Muzykantov VR. Selective targeting of nanomedicine to inflamed cerebral vasculature to enhance the blood–brain barrier. Proc Natl Acad Sci USA 2020;117:3405–3414. 32005712 10.1073/pnas.1912012117 PMC7035611

[164] Li R , Kowalski PS, Morselt HWM, Schepel I, Jongman RM, Aslan A, Ruiters MHJ, Zijlstra JG, Molema G, van Meurs M, Kamps JAAM. Endothelium-targeted delivery of dexamethasone by anti-VCAM-1 SAINT-O-Somes in mouse endotoxemia. PLoS One 2018;13:e0196976. 29763440 10.1371/journal.pone.0196976 PMC5953446

[165] Shuvaev VV , Christofidou-Solomidou M, Scherpereel A, Simone E, Arguiri E, Tliba S, Pick J, Kennel S, Albelda SM, Muzykantov VR. Factors modulating the delivery and effect of enzymatic cargo conjugated with antibodies targeted to the pulmonary endothelium. J Control Release 2007;118:235–244. 17270308 10.1016/j.jconrel.2006.12.025 PMC1855632

[166] Dahlman JE , Barnes C, Khan O, Thiriot A, Jhunjunwala S, Shaw TE, Xing Y, Sager HB, Sahay G, Speciner L, Bader A, Bogorad RL, Yin H, Racie T, Dong Y, Jiang S, Seedorf D, Dave A, Sandu KS, Webber MJ, Novobrantseva T, Ruda VM, Lytton-Jean AKR, Levins CG, Kalish B, Mudge DK, Perez M, Abezgauz L, Dutta P, Smith L, Charisse K, Kieran MW, Fitzgerald K, Nahrendorf M, Danino D, Tuder RM, von Andrian UH, Akinc A, Schroeder A, Panigrahy D, Kotelianski V, Langer R, Anderson DG. *In vivo* endothelial siRNA delivery using polymeric nanoparticles with low molecular weight. Nat Nanotechnol 2014;9:648–655. 24813696 10.1038/nnano.2014.84 PMC4207430

[167] Sago CD , Lokugamage MP, Paunovska K, Vanover DA, Monaco CM, Shah NN, Gamboa Castro M, Anderson SE, Rudoltz TG, Lando GN, Munnilal Tiwari P, Kirschman JL, Willett N, Jang YC, Santangelo PJ, Bryksin AV, Dahlman JE. High-throughput *in vivo* screen of functional mRNA delivery identifies nanoparticles for endothelial cell gene editing. Proc Natl Acad Sci USA 2018;115:E9944–E9952. 30275336 10.1073/pnas.1811276115 PMC6196543

[168] Halpern KB , Shenhav R, Massalha H, Toth B, Egozi A, Massasa EE, Medgalia C, David E, Giladi A, Moor AE, Porat Z, Amit I, Itzkovitz S. Paired-cell sequencing enables spatial gene expression mapping of liver endothelial cells. Nat Biotechnol 2018;36:962–970. 30222169 10.1038/nbt.4231 PMC6546596

[169] Aran D , Looney AP, Liu L, Wu E, Fong V, Hsu A, Chak S, Naikawadi RP, Wolters PJ, Abate AR, Butte AJ, Bhattacharya M. Reference-based analysis of lung single-cell sequencing reveals a transitional profibrotic macrophage. Nat Immunol 2019;20:163–172. 30643263 10.1038/s41590-018-0276-y PMC6340744

[170] Pasquini G , Rojo Arias JE, Schäfer P, Busskamp V. Automated methods for cell type annotation on scRNA-seq data. Comput Struct Biotechnol J 2021;19:961–969. 33613863 10.1016/j.csbj.2021.01.015 PMC7873570

[171] Shao X , Liao J, Lu X, Xue R, Ai N, Fan X. scCATCH: automatic annotation on cell types of clusters from single-cell RNA sequencing data. iScience 2020;23:100882. 32062421 10.1016/j.isci.2020.100882 PMC7031312

[172] Abdelaal T , Michielsen L, Cats D, Hoogduin D, Mei H, Reinders MJT, Mahfouz A. A comparison of automatic cell identification methods for single-cell RNA sequencing data. Genome Biol 2019;20:194. 31500660 10.1186/s13059-019-1795-z PMC6734286

[173] Hao Y , Hao S, Andersen-Nissen E, Mauck WM, Zheng S, Butler A, Lee MJ, Wilk AJ, Darby C, Zager M, Hoffman P, Stoeckius M, Papalexi E, Mimitou EP, Jain J, Srivastava A, Stuart T, Fleming LM, Yeung B, Rogers AJ, McElrath JM, Blish CA, Gottardo R, Smibert P, Satija R. Integrated analysis of multimodal single-cell data. Cell 2021;184:3573–3587.e29. 34062119 10.1016/j.cell.2021.04.048 PMC8238499

[174] Schupp JC , Adams TS, Cosme C, Raredon MSB, Yuan Y, Omote N, Poli S, Chioccioli M, Rose K-A, Manning EP, Sauler M, DeIuliis G, Ahangari F, Neumark N, Habermann AC, Gutierrez AJ, Bui LT, Lafyatis R, Pierce RW, Meyer KB, Nawijn MC, Teichmann SA, Banovich NE, Kropski JA, Niklason LE, Pe’er D, Yan X, Homer RJ, Rosas IO, Kaminski N. Integrated single cell atlas of endothelial cells of the human lung. Circulation 2021;144:286–302. 34030460 10.1161/CIRCULATIONAHA.120.052318 PMC8300155

[175] Papatheodorou I , Moreno P, Manning J, Fuentes AM-P, George N, Fexova S, Fonseca NA, Füllgrabe A, Green M, Huang N, Huerta L, Iqbal H, Jianu M, Mohammed S, Zhao L, Jarnuczak AF, Jupp S, Marioni J, Meyer K, Petryszak R, Prada Medina CA, Talavera-López C, Teichmann S, Vizcaino JA, Brazma A. Expression Atlas update: from tissues to single cells. Nucleic Acids Res 2020;48:D77–D83. 31665515 10.1093/nar/gkz947 PMC7145605

[176] Svensson V , da Veiga Beltrame E, Pachter L. A curated database reveals trends in single-cell transcriptomics. Database 2020;2020:baaa073. 33247933 10.1093/database/baaa073 PMC7698659

[177] Franzén O , Gan L-M, Björkegren JLM. PanglaoDB: a web server for exploration of mouse and human single-cell RNA sequencing data. Database 2019;2019:baz046. 30951143 10.1093/database/baz046 PMC6450036

[178] Cao Y , Zhu J, Jia P, Zhao Z. scRNASeqDB: a database for RNA-seq based gene expression profiles in human single cells. Genes (Basel) 2017;8:368. 29206167 10.3390/genes8120368 PMC5748686

[179] Ner-Gaon H , Melchior A, Golan N, Ben-Haim Y, Shay T. JingleBells: a repository of immune-related single-cell RNA-sequencing datasets. J Immunol 2017;198:3375–3379. 28416714 10.4049/jimmunol.1700272

[180] Yuan H , Yan M, Zhang G, Liu W, Deng C, Liao G, Xu L, Luo T, Yan H, Long Z, Shi A, Zhao T, Xiao Y, Li X. CancerSEA: a cancer single-cell state atlas. Nucleic Acids Res 2019;47:D900–D908. 30329142 10.1093/nar/gky939 PMC6324047

[181] Regev A , Teichmann SA, Lander ES, Amit I, Benoist C, Birney E, Bodenmiller B, Campbell P, Carninci P, Clatworthy M, Clevers H, Deplancke B, Dunham I, Eberwine J, Eils R, Enard W, Farmer A, Fugger L, Göttgens B, Hacohen N, Haniffa M, Hemberg M, Kim S, Klenerman P, Kriegstein A, Lein E, Linnarsson S, Lundberg E, Lundeberg J, Majumder P, Marioni JC, Merad M, Mhlanga M, Nawijn M, Netea M, Nolan G, Pe'er D, Phillipakis A, Ponting CP, Quake S, Reik W, Rozenblatt-Rosen O, Sanes J, Satija R, Schumacher TN, Shalek A, Shapiro E, Sharma P, Shin JW, Stegle O, Stratton M, Stubbington MJT, Theis FJ, Uhlen M, van Oudenaarden A, Wagner A, Watt F, Weissman J, Wold B, Xavier R, Yosef N; Human Cell Atlas Meeting Participants. The human cell atlas. eLife 2017;6:e27041. 29206104 10.7554/eLife.27041 PMC5762154

[182] HuBMAP Consortium . The human body at cellular resolution: the NIH Human Biomolecular Atlas Program. Nature 2019;574:187–192. 31597973 10.1038/s41586-019-1629-x PMC6800388

[183] Lee J , Yoon W, Kim S, Kim D, Kim S, So CH, Kang J. BioBERT: a pre-trained biomedical language representation model for biomedical text mining. Bioinformatics 2020;36:1234–1240. 31501885 10.1093/bioinformatics/btz682 PMC7703786

[184] Simon C , Davidsen K, Hansen C, Seymour E, Barnkob MB, Olsen LR. BioReader: a text mining tool for performing classification of biomedical literature. BMC Bioinformatics 2019;19:57. 30717659 10.1186/s12859-019-2607-x PMC7394276

[185] Marx V. Method of the Year: spatially resolved transcriptomics. Nat Methods 2021;18:9–14. 33408395 10.1038/s41592-020-01033-y

[186] Okochi Y , Sakaguchi S, Nakae K, Kondo T, Naoki H. Model-based prediction of spatial gene expression via generative linear mapping. Nat Commun 2021;12:3731. 34140477 10.1038/s41467-021-24014-x PMC8211835

[187] Ren X , Zhong G, Zhang Q, Zhang L, Sun Y, Zhang Z. Reconstruction of cell spatial organization from single-cell RNA sequencing data based on ligand-receptor mediated self-assembly. Cell Res 2020;30:763–778. 32541867 10.1038/s41422-020-0353-2 PMC7608415

[188] Nitzan M , Karaiskos N, Friedman N, Rajewsky N. Gene expression cartography. Nature 2019;576:132–137. 31748748 10.1038/s41586-019-1773-3

[189] Abdelaal T , Mourragui S, Mahfouz A, Reinders MJT. SpaGE: spatial Gene Enhancement using scRNA-seq. Nucleic Acids Res 2020;48:e107. 32955565 10.1093/nar/gkaa740 PMC7544237

[190] Cang Z , Nie Q. Inferring spatial and signaling relationships between cells from single cell transcriptomic data. Nat Commun 2020;11:2084. 32350282 10.1038/s41467-020-15968-5 PMC7190659

[191] Stoeckius M , Hafemeister C, Stephenson W, Houck-Loomis B, Chattopadhyay PK, Swerdlow H, Satija R, Smibert P. Simultaneous epitope and transcriptome measurement in single cells. Nat Methods 2017;14:865–868. 28759029 10.1038/nmeth.4380 PMC5669064

[192] Peterson VM , Zhang KX, Kumar N, Wong J, Li L, Wilson DC, Moore R, McClanahan TK, Sadekova S, Klappenbach JA. Multiplexed quantification of proteins and transcripts in single cells. Nat Biotechnol 2017;35:936–939. 28854175 10.1038/nbt.3973

[193] Spitzer MH , Nolan GP. Mass cytometry: single cells, many features. Cell 2016;165:780–791. 27153492 10.1016/j.cell.2016.04.019 PMC4860251

[194] Gault J , Liko I, Landreh M, Shutin D, Bolla JR, Jefferies D, Agasid M, Yen H-Y, Ladds MJGW, Lane DP, Khalid S, Mullen C, Remes PM, Huguet R, McAlister G, Goodwin M, Viner R, Syka JEP, Robinson CV. Combining native and ‘omics’ mass spectrometry to identify endogenous ligands bound to membrane proteins. Nat Methods 2020;17:505–508. 32371966 10.1038/s41592-020-0821-0 PMC7332344

[195] Katzenelenbogen Y , Sheban F, Yalin A, Yofe I, Svetlichnyy D, Jaitin DA, Bornstein C, Moshe A, Keren-Shaul H, Cohen M, Wang S-Y, Li B, David E, Salame T-M, Weiner A, Amit I. Coupled scRNA-seq and intracellular protein activity reveal an immunosuppressive role of TREM2 in cancer. Cell 2020;182:872–885.e19. 32783915 10.1016/j.cell.2020.06.032

[196] Specht H , Emmott E, Petelski AA, Huffman RG, Perlman DH, Serra M, Kharchenko P, Koller A, Slavov N. Single-cell proteomic and transcriptomic analysis of macrophage heterogeneity using SCoPE2. Genome Biol 2021;22:50. 33504367 10.1186/s13059-021-02267-5 PMC7839219

[197] Deng J , Ni Z, Gu W, Chen Q, Nowak WN, Chen T, Issa Bhaloo S, Zhang Z, Hu Y, Zhou B, Zhang L, Xu Q. Single-cell gene profiling and lineage tracing analyses revealed novel mechanisms of endothelial repair by progenitors. Cell Mol Life Sci 2020;77:5299–5320. 32166394 10.1007/s00018-020-03480-4 PMC11104897

[198] Zhao G , Lu H, Chang Z, Zhao Y, Zhu T, Chang L, Guo Y, Garcia-Barrio MT, Chen YE, Zhang J. Single-cell RNA sequencing reveals the cellular heterogeneity of aneurysmal infrarenal abdominal aorta. Cardiovasc Res 2021;117:1402–1416. 32678909 10.1093/cvr/cvaa214 PMC8064434

[199] Mathys H , Davila-Velderrain J, Peng Z, Gao F, Mohammadi S, Young JZ, Menon M, He L, Abdurrob F, Jiang X, Martorell AJ, Ransohoff RM, Hafler BP, Bennett DA, Kellis M, Tsai L-H. Single-cell transcriptomic analysis of Alzheimer's disease. Nature 2019;570:332–337. 31042697 10.1038/s41586-019-1195-2 PMC6865822

[200] Wirka RC , Wagh D, Paik DT, Pjanic M, Nguyen T, Miller CL, Kundu R, Nagao M, Coller J, Koyano TK, Fong R, Woo YJ, Liu B, Montgomery SB, Wu JC, Zhu K, Chang R, Alamprese M, Tallquist MD, Kim JB, Quertermous T. Atheroprotective roles of smooth muscle cell phenotypic modulation and the TCF21 disease gene as revealed by single-cell analysis. Nat Med 2019;25:1280–1289. 31359001 10.1038/s41591-019-0512-5 PMC7274198

[201] DePasquale EAK , Schnell DJ, Van Camp P-J, Valiente-Alandí Í, Blaxall BC, Grimes HL, Singh H, Salomonis N. DoubletDecon: deconvoluting doublets from single-cell RNA-sequencing data. Cell Rep 2019;29:1718–1727.e8. 31693907 10.1016/j.celrep.2019.09.082 PMC6983270

[202] Bernstein NJ , Fong NL, Lam I, Roy MA, Hendrickson DG, Kelley DR. Solo: doublet identification in single-cell RNA-seq via semi-supervised deep learning. Cell Syst 2020;11:95–101.e5. 32592658 10.1016/j.cels.2020.05.010

[203] Bais AS , Kostka D. scds: computational annotation of doublets in single-cell RNA sequencing data. Bioinformatics 2020;36:1150–1158. 31501871 10.1093/bioinformatics/btz698 PMC7703774

[204] Wolock SL , Lopez R, Klein AM. Scrublet: computational identification of cell doublets in single-cell transcriptomic data. Cell Syst 2019;8:281–291.e9. 30954476 10.1016/j.cels.2018.11.005 PMC6625319

[205] McGinnis CS , Murrow LM, Gartner ZJ. DoubletFinder: doublet detection in single-cell RNA sequencing data using artificial nearest neighbors. Cell Syst 2019;8:329–337.e4. 30954475 10.1016/j.cels.2019.03.003 PMC6853612

[206] Stoeckius M , Zheng S, Houck-Loomis B, Hao S, Yeung BZ, Mauck WM, Smibert P, Satija R. Cell hashing with barcoded antibodies enables multiplexing and doublet detection for single cell genomics. Genome Biol 2018;19:224. 30567574 10.1186/s13059-018-1603-1 PMC6300015

[207] Feng C , Liu S, Zhang H, Guan R, Li D, Zhou F, Liang Y, Feng X. Dimension reduction and clustering models for single-cell RNA sequencing data: a comparative study. Int J Mol Sci 2020;21:2181. 32235704 10.3390/ijms21062181 PMC7139673

[208] Huang M , Wang J, Torre E, Dueck H, Shaffer S, Bonasio R, Murray JI, Raj A, Li M, Zhang NR. SAVER: gene expression recovery for single-cell RNA sequencing. Nat Methods 2018;15:539–542. 29941873 10.1038/s41592-018-0033-z PMC6030502

[209] van Dijk D , Sharma R, Nainys J, Yim K, Kathail P, Carr AJ, Burdziak C, Moon KR, Chaffer CL, Pattabiraman D, Bierie B, Mazutis L, Wolf G, Krishnaswamy S, Pe'er D. Recovering gene interactions from single-cell data using data diffusion. Cell 2018;174:716–729.e27. 29961576 10.1016/j.cell.2018.05.061 PMC6771278

[210] Lytal N , Ran D, An L. Normalization methods on single-cell RNA-seq data: an empirical survey. Front Genet 2020;11:41. 32117453 10.3389/fgene.2020.00041 PMC7019105

[211] Tran HTN , Ang KS, Chevrier M, Zhang X, Lee NYS, Goh M, Chen J. A benchmark of batch-effect correction methods for single-cell RNA sequencing data. Genome Biol 2020;21:12. 31948481 10.1186/s13059-019-1850-9 PMC6964114

[212] Chazarra-Gil R , van Dongen S, Kiselev VY, Hemberg M. Flexible comparison of batch correction methods for single-cell RNA-seq using BatchBench. Nucleic Acids Res 2021;49:e42. 33524142 10.1093/nar/gkab004 PMC8053088

[213] Haghverdi L , Lun ATL, Morgan MD, Marioni JC. Batch effects in single-cell RNA-sequencing data are corrected by matching mutual nearest neighbors. Nat Biotechnol 2018;36:421–427. 29608177 10.1038/nbt.4091 PMC6152897

[214] Stuart T , Butler A, Hoffman P, Hafemeister C, Papalexi E, Mauck WM, Hao Y, Stoeckius M, Smibert P, Satija R. Comprehensive integration of single-cell data. Cell 2019;177:1888–1902.e21. 31178118 10.1016/j.cell.2019.05.031 PMC6687398

[215] Lähnemann D , Köster J, Szczurek E, McCarthy DJ, Hicks SC, Robinson MD, Vallejos CA, Campbell KR, Beerenwinkel N, Mahfouz A, Pinello L, Skums P, Stamatakis A, Attolini CS-O, Aparicio S, Baaijens J, Balvert M, Barbanson B. D, Cappuccio A, Corleone G, Dutilh BE, Florescu M, Guryev V, Holmer R, Jahn K, Lobo TJ, Keizer EM, Khatri I, Kielbasa SM, Korbel JO, Kozlov AM, Kuo T-H, Lelieveldt BPF, Mandoiu II, Marioni JC, Marschall T, Mölder F, Niknejad A, Raczkowski L, Reinders M, Ridder J. D, Saliba A-E, Somarakis A, Stegle O, Theis FJ, Yang H, Zelikovsky A, McHardy AC, Raphael BJ, Shah SP, Schönhuth A. Eleven grand challenges in single-cell data science. Genome Biol 2020;21:31. 32033589 10.1186/s13059-020-1926-6 PMC7007675

[216] Slyper M , Porter CBM, Ashenberg O, Waldman J, Drokhlyansky E, Wakiro I, Smillie C, Smith-Rosario G, Wu J, Dionne D, Vigneau S, Jané-Valbuena J, Tickle TL, Napolitano S, Su M-J, Patel AG, Karlstrom A, Gritsch S, Nomura M, Waghray A, Gohil SH, Tsankov AM, Jerby-Arnon L, Cohen O, Klughammer J, Rosen Y, Gould J, Nguyen L, Hofree M, Tramontozzi PJ, Li B, Wu CJ, Izar B, Haq R, Hodi FS, Yoon CH, Hata AN, Baker SJ, Suvà ML, Bueno R, Stover EH, Clay MR, Dyer MA, Collins NB, Matulonis UA, Wagle N, Johnson BE, Rotem A, Rozenblatt-Rosen O, Regev A. A single-cell and single-nucleus RNA-Seq toolbox for fresh and frozen human tumors. Nat Med 2020;26:792–802. 32405060 10.1038/s41591-020-0844-1 PMC7220853

[217] Sokol L , Geldhof V, García-Caballero M, Conchinha NV, Dumas SJ, Meta E, Teuwen L-A, Veys K, Chen R, Treps L, Borri M, de Zeeuw P, Falkenberg KD, Dubois C, Parys M, de Rooij LPMH, Goveia J, Rohlenova K, Schoonjans L, Dewerchin M, Eelen G, Li X, Kalucka J, Carmeliet P. Protocols for endothelial cell isolation from mouse tissues: small intestine, colon, heart, and liver. STAR Protoc 2021;2:100489. 34007969 10.1016/j.xpro.2021.100489 PMC8111824

[218] Conchinha NV , Sokol L, Teuwen L-A, Veys K, Dumas SJ, Meta E, García-Caballero M, Geldhof V, Chen R, Treps L, Borri M, de Zeeuw P, Falkenberg KD, Dubois C, Parys M, de Rooij LPMH, Rohlenova K, Goveia J, Schoonjans L, Dewerchin M, Eelen G, Li X, Kalucka J, Carmeliet P. Protocols for endothelial cell isolation from mouse tissues: brain, choroid, lung, and muscle. STAR Protoc 2021;2:100508. 34585146 10.1016/j.xpro.2021.100508 PMC8450255

[219] Dumas SJ , Meta E, Conchinha NV, Sokol L, Chen R, Borri M, Teuwen L-A, Veys K, García-Caballero M, Geldhof V, Treps L, de Zeeuw P, Falkenberg KD, Dubois C, Parys M, de Rooij LPMH, Rohlenova K, Goveia J, Schoonjans L, Dewerchin M, Eelen G, Li X, Kalucka J, Carmeliet P. Protocols for endothelial cell isolation from mouse tissues: kidney, spleen, and testis. STAR Protoc 2021;2:100523–100523. 34382011 10.1016/j.xpro.2021.100523 PMC8339245

